# Climate change could threaten cocoa production: Effects of 2015-16 El Niño-related drought on cocoa agroforests in Bahia, Brazil

**DOI:** 10.1371/journal.pone.0200454

**Published:** 2018-07-10

**Authors:** Lauranne Gateau-Rey, Edmund V. J. Tanner, Bruno Rapidel, Jean-Philippe Marelli, Stefan Royaert

**Affiliations:** 1 Department of Plant Sciences, University of Cambridge, Cambridge, United Kingdom; 2 CIRAD, UMR SYSTEM, Montpellier, France; 3 UMR SYSTEM, University of Montpellier, CIRAD, INRA, Montpellier SupAgro, Montpellier, France; 4 MARS/USDA Cocoa Laboratory, Miami, Florida, United States of America; 5 MARS Center for Cocoa Science, Itajuípe, Bahia, Brazil; The University of Auckland, NEW ZEALAND

## Abstract

Climate models predict a possible increase in the frequency of strong climate events such as El Niño-Southern Oscillation (ENSO), which in parts of the tropics are the cause of exceptional droughts, these threaten global food production. Agroforestry systems are often suggested as promising diversification options to increase farmers' resilience to extreme climatic events. In the Northeastern state of Bahia, where most Brazilian cocoa is grown in wildlife-friendly agroforests, ENSOs cause severe droughts which negatively affect forest and agriculture. Cocoa (Theobroma cacao) is described as being sensitive to drought but there are no field-studies of the effect of ENSO-related drought on adult cocoa trees in the America's; there is one study of an experimentally-imposed drought in Indonesia which resulted in 10 to 46% yield loss. In our study, in randomly chosen farms in Bahia, Brazil, we measured the effect of the 2015–16 severe ENSO, which caused an unprecedented drought in cocoa agroforests. We show that drought caused high cocoa tree mortality (15%) and severely decreased cocoa yield (89%); the drought also increased infection rate of the chronic fungal disease witches' broom (Moniliophthora perniciosa). Ours findings showed that Brazilian cocoa agroforests are at risk and that increasing frequency of strong droughts are likely to cause decreased cocoa yields in the coming decades. Furthermore, because cocoa, like many crops, is grown somewhat beyond its climatic limits, it and other crops could be the 'canaries in the coalmine' warning of forthcoming major drought effects on semi-natural and natural vegetation.

## Introduction

Climate change is likely to affect global food production [1–3]. Agriculture is threatened by extreme climatic events such as droughts or floods enhanced by climate change [4]. There is still an active discussion and a high uncertainty about the impact of climate change on ENSO frequency and intensity. Some climate change scenarios predict an increase in extreme events, including an increased frequency of strong El Niño Southern Oscillation (ENSO) events [5–7], which cause drought and flooding in the tropics. However, other studies did not predict an increase in frequency of ENSO events associated with climate change [8,9]. Starting in October 2014 and lasting until May 2016 there was a strong ENSO event, it was responsible for severe droughts in North-eastern Brazil [10] where previous ENSO-related droughts have affected forest cover [11–13] and agricultural yields [14,15].

Brazil is the largest cocoa producer in South America with an average production of 200,000 tonnes of dry cocoa beans in 2014–2015 [16], 75% of which was produced in Bahia. Cocoa is usually grown under the shade of large trees, which are a mixture of native species from the Atlantic Rainforest and introduced species grown for food (such as native *Spondias mombin* and exotic *Artocarpus heterophyllus*), timber (such as native *Cariniana legalis*) or nitrogen fixation (such as native *Plathymenia foliosa* and exotic *Erythrina spp*). These agroforestry systems, called cabrucas in Bahia [17,18] are a type of crop diversification commonly found in the tropics. Such diversifications are often suggested to increase farmers’ resilience to extreme climatic events [19–21]. However, there is no field-based study of the effect of ENSO-related drought on cabrucas despite the importance of cocoa as a crop in Bahia and the frequent droughts experienced in the region—on average every 6 years-though with much variation [22].

Cocoa is described as being sensitive to drought [23], but there are few field-studies on the effect of drought on cocoa. Published studies of cocoa bean yields and their decrease due to ENSO-related droughts are based on interviews with farmers and/or official national statistical data. In Sulawesi, it was found that ENSO-related drought caused a 62% loss of cocoa production compared to their usual levels—based on data provided by the farmers [24]. In West Africa 27% loss compared to a normal year was reported [25], but this loss was mainly due to a decrease in the planted area due to forest fires, it was not much caused by lower production in drought affected cocoa trees. In Ecuador, 19% loss of cocoa planted area was found as a result of the 1997–98 ENSO [26]. A physiological production model compiling data from 30 sites in 10 cocoa producing countries, concluded that water limitation was responsible for 50% loss in yield [27]. A climate change model for West African cocoa production [28,29] predicted that the possible decrease in area suitable for growing cocoa by 2050 was mainly due to increased temperature and surprisingly not due to a decrease in rainfall. Overall these reports show that many areas have strong reductions in cocoa production due to drought though none of the studies is based on detailed research of the effects of drought on cocoa trees on farms.

The only large detailed on-farm study of the effects of drought on cocoa is of an experimentally imposed drought on six-year old cocoa grown with six-year old Gliricidia shade in Indonesia [30–34]. The c. 78% rainfall exclusion over 13 months (of about 3000 mm rain in that period) caused only a 10% loss in cocoa yield during the rainfall exclusion, though a further 45% reduction was recorded after the end of the drought; no cocoa tree mortality was observed.

Our study measured the effect of a severe ENSO-related drought on cocoa trees in randomly chosen farms in the traditional cocoa producing area in the Northeast of Brazil, where 75% Brazilian cocoa is grown. The region has been affected by severe ENSO-related droughts in the past, however the severity of 2015–16 ENSO-related drought was unprecedented. Our major concern is that ENSO-related droughts are threatening cocoa production in traditional agroforests in the area in the long-term. We used permanent transects to measure the effect of a very strong drought on cocoa trees in 31 randomly-chosen farms with traditional cocoa agroforestry systems. To our knowledge, this is the first on-farm study of the effect of a severe natural drought on adult cocoa trees where cocoa was compared before and after an ENSO event. We expected to find reductions in cocoa yield and thus that predicted increases in drought are likely to cause major reductions in cocoa yield in the coming decades as the climate changes and strong droughts increase. Forest drought has recently emerged as a research priority [35]; drought effects on cocoa agroforestry could be a ‘canary in the coal mine’ warning of problems to come both in agriculture and in semi-natural and natural vegetation due to increased intensity and frequency of droughts in a changing world climate.

## Materials and methods

### Study site

The study area was in the municipality of Barro Preto in the south of Bahia State, Brazil (14.05° S, 39.040°W) at 150 m a.s.l. The climate is Af in the Köppen classification [36]. Annual rainfall average is 1608 mm per year with May and September being the driest months, with respectively 110 mm and 67 mm (2001 to 2014 average data from Mars Center for Cocoa Sciences (MCCS) weather station). In Barro Preto, rainfall quantities and distribution pattern are almost at the limits for cocoa production. Mean annual temperature is 26°C. Cocoa flowering follows the seasonal rainfall pattern with peaks immediately after rain events. Bahian cocoa production normally has two harvests per year: the main harvest is from April to August., there is a secondary harvest is from November to February. The soils in Barro Preto are classified as Latosols or Argisol Red-Yellow soils, they have a clayey-loam composition [37].

We selected Barro Preto municipality because of its location in the centre of the historical cocoa producing region and because of its proximity to the MCCS. The municipality has a forest cover of approximately 80% [38,39]. Cocoa farms cover 9,100 ha, more than half of the total area of the municipality (16,000 ha). Traditional cocoa agroforests, called cabrucas, have tree species both native from the Atlantic Forest ecosystem and introduced of economic interest (i.e. fruits, timber and rubber) with cocoa as the main cash crop.

Management practices are usually limited to harvesting and occasionally pruning and manual weeding. Fertiliser and pesticides use are unusual. Most farms use herbicides (Glyphosate) to delay and reduce regrowth after manual weeding. During the study, manure fertilisation was applied in only 2 of the 31 farms. Cocoa trees were pruned annually after the main harvest in most farms.

### Experimental design

From the 333 traditional cocoa farms listed in CEPLAC (Executive Commission of the Cocoa Farming Plan, the government organisation part of the ministry of agriculture in charge of research and technical support for Brazilian cocoa production) rural census for Barro Preto, we chose 31 at random, amounting approximately 1760 ha of the area planted with cocoa. In March 2015, we established permanent transects of 8 m x 100 m in areas defined as representative of each farm by the farm administrator. GPS coordinates of the transects were recorded. All cocoa trees and woody shade trees > 1.5 cm of DBH (> 5 cm of circumference) at 1.3 m above ground were measured and identified with tags. Cocoa tree and shade tree densities were on average 622 (± 33 SE) and 126 (± 12 SE) per hectare respectively. Shade level was estimated by measuring the gap fraction (and the inverse ground cover) using hemispherical photos: one photo was taken every 10 m along the 100 m transect, above the cocoa tree but below the shade tree canopy to measure average percentage of ground cover per transect. We used an EOS 5D Nikon camera with a hemispherical lens, attached to a telescopic gimbal. All photos were analysed using Canopy analyser HEMIv9–HemiView, delta-T, Cambridge, UK.

### Rain, PET data, and drought index

Rainfall and temperature data were recorded at MCCS weather station from January 2001 to February 2017. All farms were located within 15 km of MCCS. The PET was calculated from temperature using FAO ET0 calculator Version 3.2, September 2012 [40]. This calculator, based on the Penman-Monteith equation, uses average, minimum and maximum day temperature as minimum data inputs. (Average PET/month _2001–2016_ = 111 mm). We calculated the sum of average rainfall for the 30 preceding days to compare it with the sum of PET for the 30 preceding days. When the sum of the 30 preceding days of rain was less than the sum of the 30 preceding days of PET, we considered that the cocoa trees where facing a drought event. We compared water balance (rain, PET and soil water holding capacity) for a two-year period during ENSO (March 2015 to February 2017), which was the worst two-year drought since 2001 including the ENSO of 2008 (May 2007 to April 2009). The severity of the 2015–16 drought was also assessed using the widely used Standardized Evapotranspiration Precipitation Index (SPEI), calculated from the SPEI package in R [41], and 15 years of MCCS rainfall as input data. We used the database from National Oceanographic and Atmospheric Administration (NOAA) to identify the duration of El Niño-Southern Oscillation (ENSO) events and to classify their strength using the Oceanographic El Niño Index (ONI), based on anomalies in Sea Surface Temperature (SST): weak (0.5 to 0.9°C SST anomaly), moderate (1 to 1.4°C SST anomaly), strong (1.5 to 1.9°C SST anomaly) and very strong (>1.9°C SST anomaly).

### Soil water holding capacity

We measured soil water holding capacity in the middle of each transect in 10 of the 31 farms in April 2017. Because of limited resources and time, we could not make measurement in all 31 farms. In situ soil had its roots cut (in the top 5 cm of soil) was saturated with water one day and samples were collected the next day from the top 60 cm (0–20, 20–40, 40–60 cm). Samples were fresh weighed and then dried for 3 days at 90°C and reweighed. The difference in weight allowed us to calculate the water content, (m_water_ = m_wet_-m_dry_, with m_water_: mass of water, m_wet_: mass of wet soil and m_dry_: mass of dry soil). This mass of water was converted into water depth (mm), at field capacity using the formula h = V/ (π x r^2^) with h: water height in the soil core in mm, V = volume of water in mm^3^ and r = radius of soil core (15 mm). One of the ten farms was excluded because the soil was extremely rocky.

### Tree mortality

The number of dead cocoa trees was recorded in the middle of the second drought in April 2016 and compared to the number of live cocoa trees recorded in April 2015. Dead trees were recorded again in November 2017 and additional mortality was marginal (1.7%). The mortality rate of the cocoa trees (*m*) is given by *m* = 1-[(N_0_-N_m_)/N_0_]^1/t^ where N_0_ is the number of trees at the beginning of the interval, N_m_ the number of trees that died after one year (time t = 1 year). Cocoa trees were classified into 3 groups by MCCS technicians based on field characteristics: 1) ‘common’—Amazonian Amelonado including Marañon and Para the varieties most commonly found in Bahia; 2) ‘clones’, grafted or rooted cuttings of identified clones such as CCN51, TSH1188, PH16 or PS1319 selected by research institutes; and 3) hybrids, seed material produced in the 1960’s by CEPLAC mainly from hybridization of the ‘clones’ and other varieties.

### Cocoa yield and pod loss due to disease

The number of cocoa pods on tree counted before the bi-annual harvests in the transects was used as a proxy to estimate an average potential yield per harvest. The number of cocoa pods (length > 10 cm to exclude numerous small fruits that fall before reaching 10 cm) was counted on each tree before the harvest by the farmers in April 2015, April 2016, November 2016 and April 2017. Pod number on trees in the 800 m^2^ transects was scaled up to one hectare to calculate an average potential yield in kg/ha for each farm. (We considered that the cocoa and shade tree densities within the transects were representative of the farms). Potential yield was estimated using a conversion factor of 40 g of dry cocoa beans per healthy cocoa pod before the drought and 23 g of dry cocoa beans per healthy cocoa pod during after the drought (a relationship established for cocoa in the area). Both averages were calculated based on ripe cocoa pod sampling from Barro Preto farms respectively before the drought (May 2012) and at the end of the drought (May 2016). Approximately 30 pods were opened, fresh beans were fermented 5 days, sun-dried for about a week before being weighted.

The fungal infection rate (mainly caused by *Moniliophthora perniciosa* but also infrequently by *Phytophthora palmivora*) was assessed by recording the number of rotten pods on each tree. *M*. *perniciosa* primarily affects the leaves and vegetative development, but also impacts directly cocoa pods. The November 2015 harvest was not recorded for logistic reasons. Potential losses due to drought and disease were calculated by comparing the number of pods counted during harvests during and after the drought with the number of pods counted during harvest before the drought.

### Data analyses

Mean pod numbers for each transect were calculated for each date. Generalized mix effect models were used to explore relationships between tree mortality and individual tree and farm variables (farm characteristic: longitude and soil water holding capacity; shade: Ground cover and sum of shade trees DBH per transect). All statistical analyses were computed using R Stat version 3.4.1.

## Results

### ENSO, rainfall, PET and drought index

**Fig 1:**
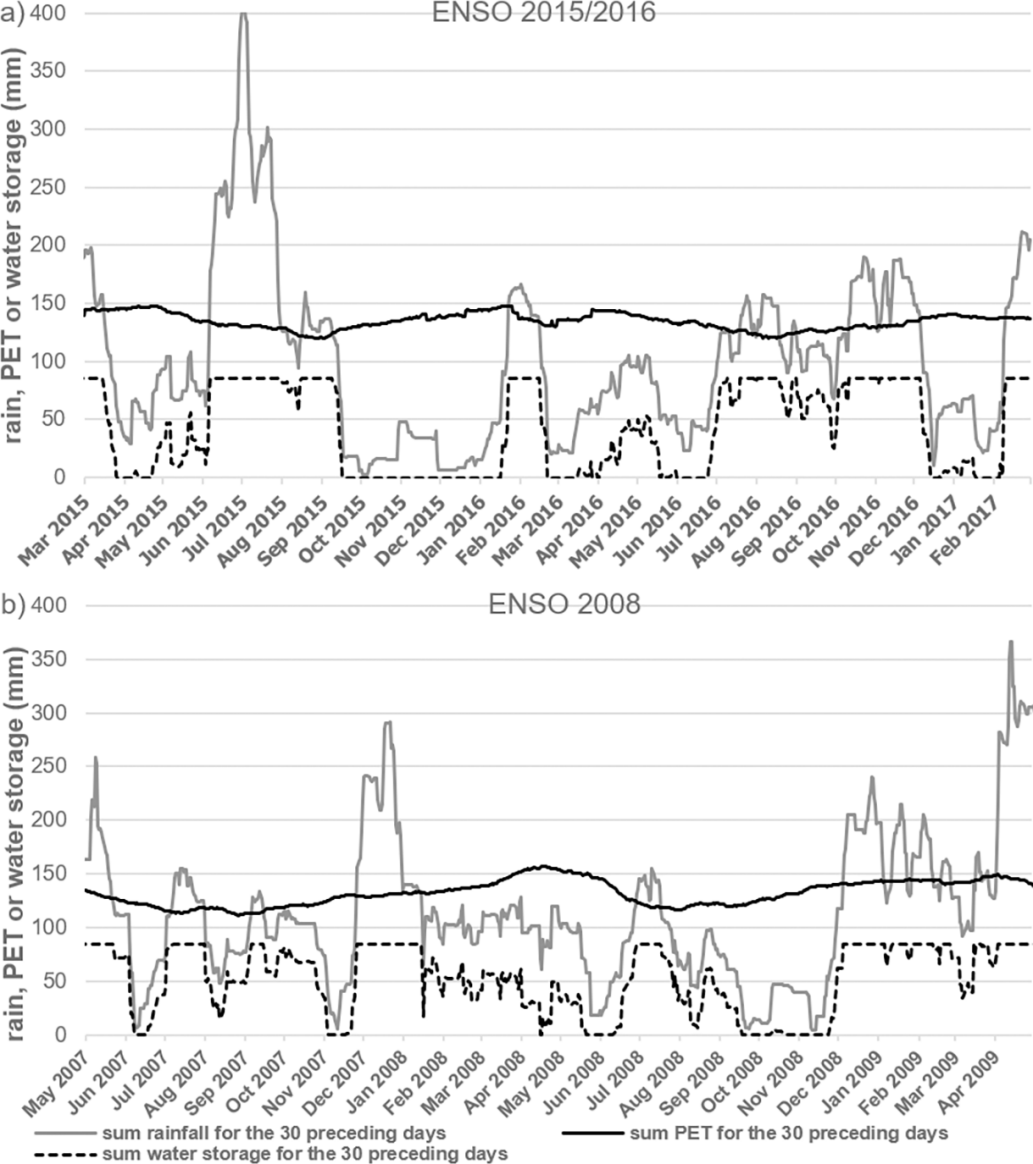
Rainfall, PET and average soil water content during ENSO. Sum of 30 preceding days in mm during ENSO 2015–16 event (a) and during ENSO 2008 (worse scenario since 2001) (b). Black line represents PET based on temperature, dark grey line represents rainfall and dotted line represents soil water storage.

When the study began in March 2015, NOAA reported an Oceanic el Niño Index (ONI) anomaly of > +0.5°C in Sea Surface Temperature (SST). ONI reached a maximum of +2.3°C in December 2015 and was > +0.5°C for 16 consecutive months until May 2016. The ENSO 2015–16 caused an abnormally strong drought in the cocoa region of Bahia in Brazil. Rainfall between August 2015 and August 2016 was 770mm (and 786 mm between August 2016 and May 2017); this is 53% lower than the 1621 (±71) mm yearly average for 14 years from August 2001. The months with the minimum rainfall were September and November 2015 with 6 and 7 mm per month respectively (Fig 1).

**Fig 2:**
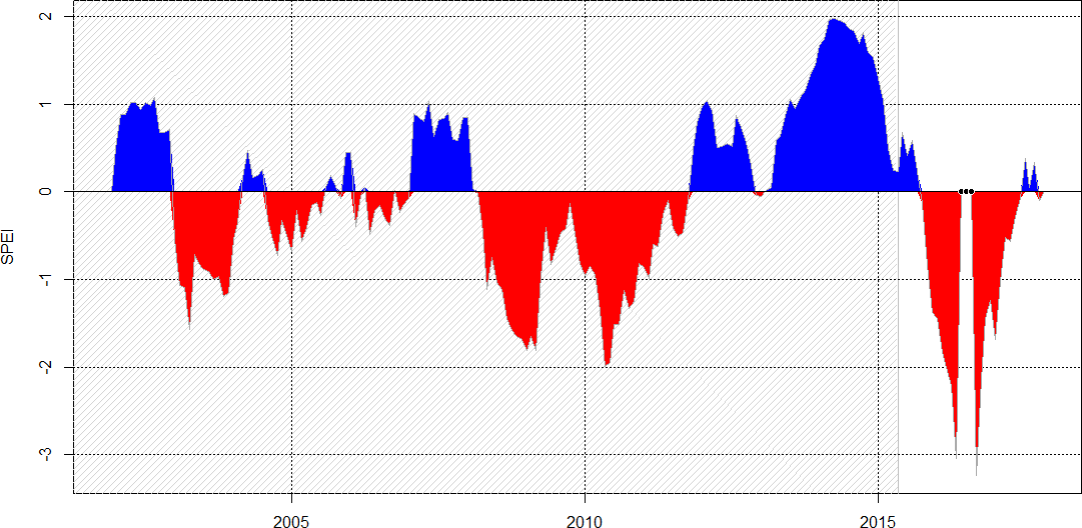
Standardized precipitation-evapotranspiration index on 12-months base. SPEI is expressed in units of standard deviation from the long-term mean of the standardized distribution. Negative values in red represent drought events. The reference period is the dashed area before ENSO.

Comparing the 30-day rainfall totals with the Potential Evapotranspiration (PET) (Fig 1) showed two very long episodes, separated by 25 days, when PET > rain: September 2015 to January 2016 (136 days) and February 2016 to end of June 2016 (131 days). From July 2016 rainfall returned to approximately equal PET–the average situation except for December 2016 until February 2017, when PET exceeded rainfall (Fig 1). Expressing the same rainfall data in a different way as the ‘standardized precipitation-evapotranspiration index’ (SPEI) showed two episodes of negatives values during ENSO 2015–16 including 4 months with SPEI< -2 and with an extreme deviation of -3.2 in November 2015 (Fig 2). These are exceptional values, SPEI was <- 2 during only 4 months in the past 168 months (14 years). More recently the lowest values for SPEI have been decreasing, indicating stronger droughts since 2001.

### Soil water holding capacity

Soil water holding capacity was about 86 (±13 SE) mm in the top 1000 mm of soil (Fig 1), it was higher in the West than the East (R^2^ = 0.63, *p* < 0.01). During the ENSO-related drought, calculated soil water content was at about zero for 4 months from mid-September 2015 to mid-January 2016 (Fig 1).

### Tree mortality

Cocoa tree mortality was 15% (±2.3 SE) during the ENSO event as compared to <1% normally (Bastide et al. 2008). In a further 5% (±1.2 SE) of the trees the large productive stems died but suckers remained alive. Cocoa mortality differed between groups 28% in hybrids, 22% in ‘common’ cocoa and 15% in ‘clones’ (χ^2^ = 53.2, 2 d.f., *P*<<0.001). There was no relationship between shade tree density, either numbers or total basal area, and cocoa mortality (Table 1). Shade tree mortality during the drought was 7% of the 317 woody shade trees on the transects; no species of shade trees was particularly affected. There were also 337 banana trees before the drought in March 2015 but these normally have a short lifespan (6 to 14 months), so it is not possible to say how many died of drought.

**Table 1:** Relationship between cocoa tree mortality and environmental factors.

Model	df	F stat	R^2^	Pvalue
log(PerDeadTree)~1				
log(PerDeadTree)~SoilWater	7	7.54	0.52	0.03*
log(PerDeadTree)~longitude	29	1.85	0.06	0.18
log(PerDeadTree)~GroundCover	22	0.3	0.014	0.59
log(PerDeadTree)~SumShadeTreeDBH	29	0.49	0.017	0.81
log(PerDeadTree)~NumberShadeTree	29	0.06	0.002	0.81
log(PerDeadTree)~SoilWater + longitude	6	3.31	0.52	0.11
log(PerDeadTree)~SoilWater + GroundCover	6	6.38	0.68	0.03*
log(PerDeadTree)~SoilWater + SumShadeTreeDBH	6	4.06	0.58	0.08
log(PerDeadTree)~SoilWater + NumberShadeTree	6	3.25	0.52	0.11
log(PerDeadTree)~SoilWater + longitude + GroundCover	5	3.8	0.69	0.09
log(PerDeadTree)~SoilWater +SumShadeTreeDBH + GroundCover	5	4.28	0.72	0.08
log(PerDeadTree)~SoilWater +NumberShadeTree + GroundCover	5	3.7	0.69	0.10
log(PerDeadTree)~SoilWater +SumShadeTreeDBH + NumberShadeTree	5	2.3	0.58	0.19
log(PerDeadTree)~SoilWater +longitude+SumShadeTreeDBH	5	2.59	0.61	0.17
log(PerDeadTree)~SoilWater +longitude+GroundCover+SumShadeTreeDBH	4	2.58	0.72	0.19

Stepwise regression between the logarithm of the tree mortality per transect (PerDeadTree) and environmental variables such as shade trees (number: NumberShadeTree and sum of Area at breast height: SumShadeTreeDBH per transect, shade tree cover: GroundCover) and farms characteristic (longitude and soil water holding capacity: SoilWater).

* Significance code: pvalue < 0.05

### Pod loss

**Fig 3:**
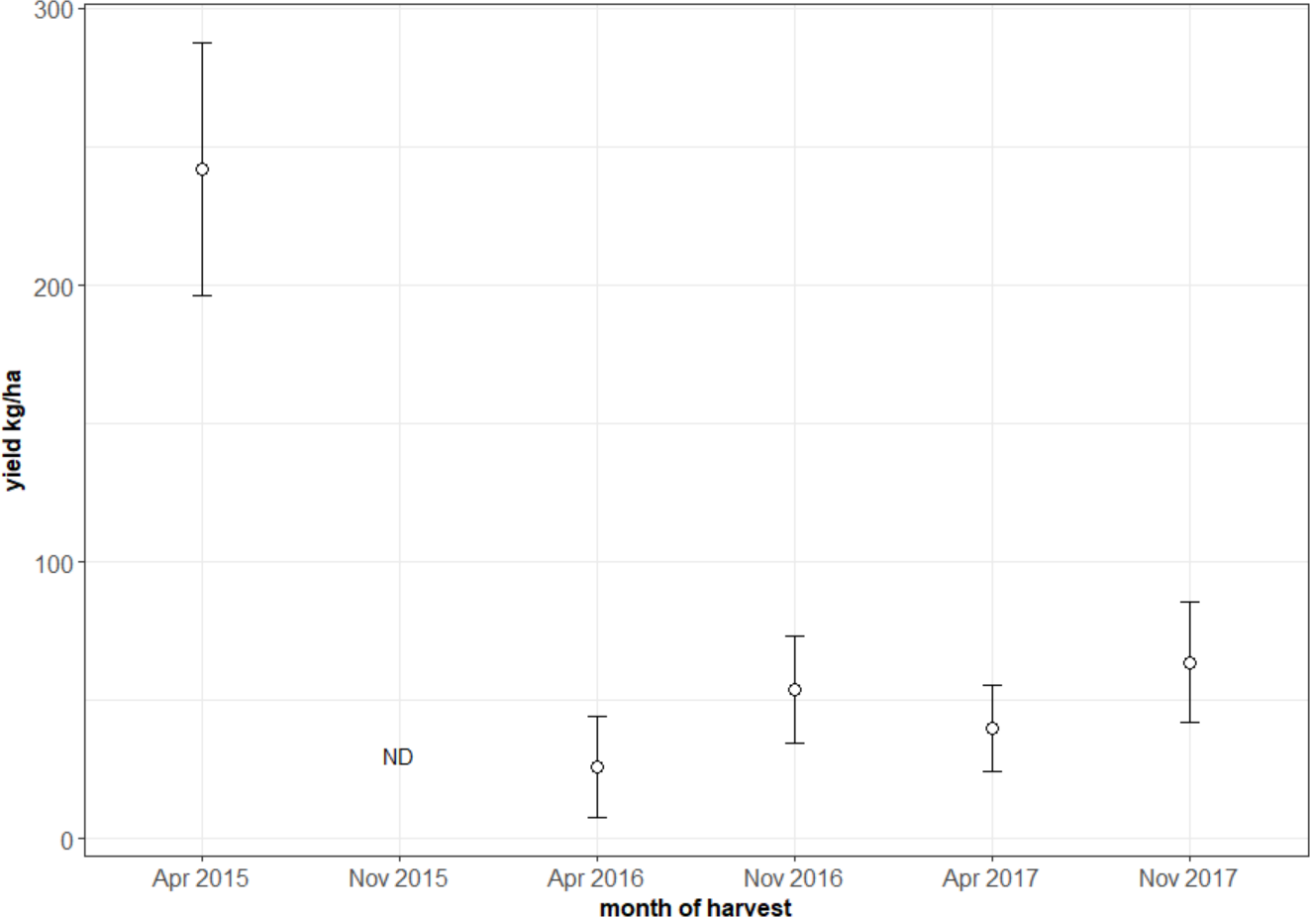
Yield per farm based on the number of pods per main harvest. The drought decreases the pod production on a long term. There was no data (ND) for the peak harvest in November 2015.

The average potential yield per area (based on pod number) on the 31 farms, for the main harvest (of two per year) in April 2015 before the drought, was 242 (± 25) kg/ha. During the drought, in April 2016, the average potential yield was 26 (± 9) kg/ha—an 89% reduction (Fig 3). Nine months after the drought ended in July 2016, the potential yield in April 2017 was still 83% lower than in April 2015. The drought dramatically decreased the number of pods per tree. The 15% mortality of productive cocoa trees caused 11% of the 89% loss.

There was no relationship between soil water holding capacity and yield loss. There was an effect of farm location (longitude) on yield loss (Table 2).

**Table 2:** Relationship between cocoa yield loss and environmental factors.

model	df	F stat	R^2^	*P*value
YieldLoss~1				
YieldLoss~SoilWater	6	3.45	0.36	0.11
YieldLoss~longitude	28	4.17	0.13	0.05
YieldLoss~GroundCover	28	0.22	0.01	0.64
YieldLoss~SumShadeTreeDBH	28	2.87	0.09	0.10
YieldLoss~NumberShadeTree	28	0.75	0.03	0.39
YieldLoss~PercentageDeadTree	28	0.65	0.02	0.43
YieldLoss~longitude + SoilWater	5	5.22	0.68	0.06
YieldLoss~SoilWater + GroundCover	5	2.21	0.47	0.21
YieldLoss~longitude + GroundCover	27	1.91	0.13	0.17
YieldLoss~longitude + SumShadeTreeDBH	27	3.51	0.14	0.04*
YieldLoss~longitude + NumberShadeTree	27	2.13	0.14	0.14
YieldLoss~longitude + SumShadeTreeDBH + SoilWater	4	3.1	0.7	0.15
YieldLoss~longitude + NumberShadeTree + GroundCover	26	1.31	0.14	0.29
YieldLoss~longitude + SumShadeTreeDBH + NumberShadeTree	26	4.24	0.33	0.01*
YieldLoss~longitude + SumShadeTreeDBH + GroundCover	26	2.17	0.21	0.12
YieldLoss~longitude + SumShadeTreeDBH + NumberShadeTree + GroundCover	24	2.84	0.32	0.05

* Significance code: pvalue < 0.05

### Disease and infection rate

Witches’ broom was first recorded in Bahia in 1989 and has resulted in a big reduction of cocoa production since. At the beginning of the study (April 2015), the fungal infection caused about a 15% pod loss. However, during the drought the loss was much higher: 36% in April 2016 and 35% in April 2017 after the drought.

### ENSO-related drought and yield loss at Brazil scale

**Fig 4:**
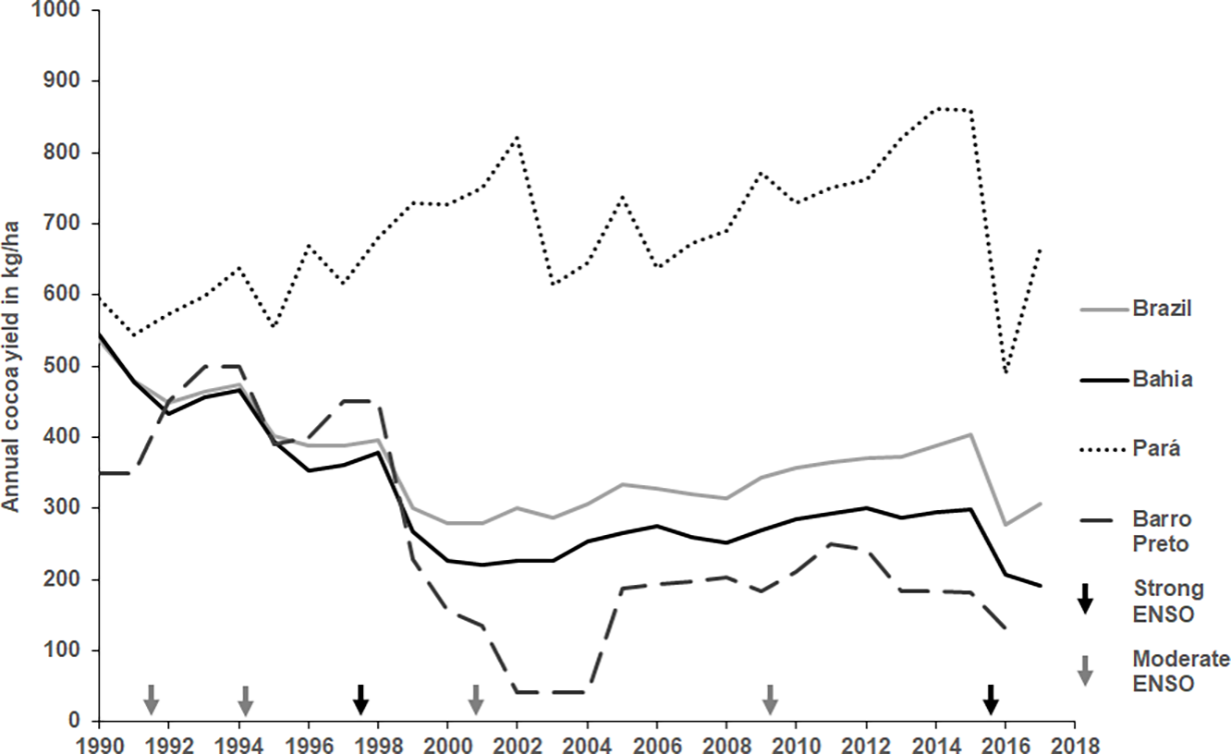
Cocoa production in Brazil, Bahia, Para and Barro Preto since 1990. The continuous grey line represents Brazilian cocoa production; the continuous black line represents the cocoa production for the state of Bahia and the dotted black line the state of Para. The discontinuous black line represents the cocoa production for the municipality of Barro Preto (source: IBGE). Black arrows represent strong ENSO events (1997–1998, 2015–2016), grey arrows represent moderate ENSO events (1991–1992, 1994–1995, 2002–2003, 2009–2010). The Moniliophthora perniciosa outbreak started in Bahia in 1989.

Strong ENSO-related droughts decreased production in Bahia and Brazil as a whole (Fig 4), though weak ENSO events did not.

Cocoa production in 2015–16 during the ENSO-related drought (277 kg ha^-1^) was the lowest recorded since 1990. At a country scale yield decreased from 400 kg ha^-1^ before the drought to 277 kg ha^-1^ after; in Para state yield decreased from 860 kg ha^-1^ to 490 kg ha^-1^, in Bahia state yield decreased from 300 kg ha^-1^ to 200 kg ha^-1^. Production for Barro Preto was not available for 2016–2017).

## Discussion

### First field data with quantification of drought sensitivity of cocoa agroforests

To our knowledge, our study on the effect of drought on cocoa agroforests, is the first, in South America, based on natural drought and field assessments on farms. Despite cocoa being known as a drought-sensitive crop [23] published, reliable, field-data on the effect of natural drought on mature plantations is scarce [42]. There seems to be only one other on-farm study of the effect of 2015/16 ENSO drought on cocoa trees, done in Ghana [43].The authors concluded that full-sun plantations were more resilient to drought than agroforests by comparing mortality, transpiration rates and soil water content in cocoa trees under only three specific shading regimes (full sun cocoa, cocoa-*Albizia ferruginea* and cocoa-*Antiaris toxicaria*) in only one farm. The generality of this conclusion have been questioned by [44,45] who pointed out that 1) these two cocoa-shade tree associations were not representative of an agroforest and 2) the sub-optimal climate of the region (based on a single site) was not representative of climate conditions where cocoa is usually grown. Thus, our study is the first recording the effect of a natural severe drought on shaded cocoa in complex agroforestry systems based on data from several cocoa farms.

In the only on-farm experiment on drought in Indonesia, plastic roofs were used over an eight-year cocoa plantation shaded by *Gliricidia*, to create a 13-month artificial drought [32]. Most cocoa roots (>83%) were found in the 40 upper cm of soil whereas *Gliricidia* roots were found evenly distributed deeper into the soil (up to 250 cm depth). Water uptake for cocoa occurred in the first 30 upper cm of soil whereas water uptake for *Gliricidia* occurred from depths >30 cm [30]. In the roof plots, the upper soil layers showed a rapid decrease in soil water content. Thus, near-surface cocoa roots were more exposed to drought than deeper *Gliricidia* roots. The difference in the distribution of roots could explain the high mortality rate in cocoa trees and low mortality of other tree species in our experiment.

Studies of the effect of previous ENSO events on cocoa plantations at regional scales in Asia, Africa and America were based on indirect data resulting from interviews and/or national statistical compilations of yield and planted area [25,26,46,47]. Our on-farm results confirm that cocoa is sensitive to very strong droughts, but the Brazilian yield data suggest that cocoa is not sensitive to mild droughts caused by weak, moderate or strong ENSO events; only ‘very strong’ ENSOs reduce yield but they do so very markedly.

### 2015–16 ENSO was the strongest event recorded over the past decades

Despite large uncertainty [9], severe ENSO events are expected to increase in frequency following climate change [5,7,48]. Droughts are not unusual in Bahia and the probability of extreme droughts in Northern Brazil is one year in nine [49]. We showed that recent ENSO was associated with the highest ONI values since the 1997–98 ENSO. It caused the strongest drought episode recorded for the last 15 years in Barro Preto, Bahia. At the global scale the ENSO 2015–16 is considered as the strongest event in the last 23 years with an SST anomaly of 0.3°C more than the highest anomaly recorded during strong 1997–98 ENSO [50]. Based on satellite data, northern Brazil and the Amazon were dramatically affected by the severe drought related to 2015–16 ENSO [10,51]. Northern Brazil had the maximum negative correlation between Vegetation health indices (VHI) and SST (−0.70) showing that vegetation was experiencing very high stress at large scale [50]. Thus, both natural vegetation and a major tree crop, cocoa, were affected by the same exceptional ENSO-related drought.

### Drought effect on yield

Potential yield losses in 2016 and 2017 were about 89% compared to the harvest in April 2015 before the drought. The decreases are much higher than the decrease in annual cocoa yield reported for the state of Bahia from 2015–2016 (about 30%: 298 to 207 kg. ha^-1^). The difference could be due to one of several, non-mutually exclusive, reasons. Firstly, our farms may have been unusually affected by the drought as compared to the rest of the state of Bahia; we cannot test this but point out that our farms were a random sample from 333 farms in the Barro Preto region (160 km^2^) however this is only 0.5% of the cocoa region of the State of Bahia (32,000 km^2^) and though the area sampled was large (16 km West to East and 10 km North to South) it is small compared to the ‘cocoa area’ of Bahia. Secondly, it could be that in wetter parts of the cocoa region of Bahia (i.e. in the North: Salvador) the ENSO-related drought had little effect or even increased growth because in wetter areas, the potential reduction in yield due to some drought may be overwhelmed by the increased production resulting from increased solar radiation. Thirdly, drought may cause abnormally high leaf loss in shade trees, which has two effects it potentially somewhat reduces water use by the shade trees and it also lets more light through to the cocoa trees below, thus increasing yield (a situation found in liana seedlings in semi-evergreen rain forest in Panama where a stronger dry season resulted increased seedling growth, [52]).

There seems to be only one other on-farm study of the effect of drought on cocoa yield—an experimental drought, simulating an ENSO event, in Indonesia, where rooves reduced rainfall by about 78% over 13 months, when the actual rainfall was 2937mm. In this experiment, there were no extended periods without rain and the ‘relative extractable water’ from the soil only reached close to zero for one month near the end of the experimental drought. Cocoa yield was only reduced by 10%, though interestingly yield was reduced by 45% *after* the rooves were removed; there was no cocoa tree mortality [30]. By contrast, in our study of natural drought we had an 89% lower yield and a 15% cocoa tree mortality; the large differences between the studies were probably due to the fact that our natural drought was much stronger (136 and 131 days with PET > rain versus 32 and 60 days under the shelters in the Indonesian experiment).

The direct effect of the drought (low water availability in soil) was to reduce the cocoa pod production and to kill many cocoa trees. Additionally, the drought could have had indirect effects on cocoa yield which were not measured during the experiment. For example, the drought could have affected the cocoa pollinator populations causing an indirect reduction in cocoa yield. In the artificial drought experiment in Indonesia, the results of hand-pollinations were not affected by the drought. However, pollination intensity was a main factor limiting yield in both roof and control plots [33]. In Bahia, the prolonged ENSO drought could have affected pollinator breeding and habitats, reducing the pollination intensity and thence indirectly reducing the cocoa yield. Furthermore, the drought could have modified the organic matter decomposition on cabruca soil. This could have limited the access to nutrients for the cocoa trees, indirectly reducing their yield.

In addition to the direct and indirect effects of drought reducing cocoa tree growth and pod production, drought was associated with an increase in disease infection rate (15% of all pods before the drought to 30% during the drought). Rotten pods resulting from disease contributed significantly (35% of the total April 2017 harvest) to the reduction in the number of pods counted after the drought. This increase in infection rate was also observed in the artificial drought experiment in Indonesia [30]. Pathogen cycles have been observed for fungal diseases for cocoa (black pod, *P*. *palmivora*) in Bahia [53] and in Nigeria [54]. Fungal disease is the major cause of cocoa yield loss worldwide being responsible for a 30% loss of production. Climate events including droughts increase these fungal infections rate and increase yield losses [55], thus putting cocoa production at risk.

### Cocoa tree mortality

Cocoa tree mortality, 15%, was exceptionally high for reasonably healthy old cocoa plantations, where normal annual tree mortality is usually <1% [56,57]. High tree losses during droughts are often caused by fire, but fire did not affect any cocoa plantations in Barro Preto during the study. However, 2,000 ha of cabrucas and forest burnt in the neighbouring municipalities as a result of the drought (reported in Intituto Arapyau: http://www.arapyau.org.br blog/2016/01, online access: 25-07-2017). Exceptionally high infection rates of pests and diseases can also be responsible for high tree mortality, for example a change from heavily shaded agroforests to non-shaded systems resulted in insect attack in São Tome and Fernando Pó in the 1920’s [58] and Vascular Streak Dieback disease *(Oncobasidium theobromae*) in Malaysia in the 1990’s [59]. No lethal pests or diseases were observed in our experiment (*Moniliophthora perniciosa* does not kill trees). The high mortality of mature cocoa trees caused by drought is important because it will reduce yields for a minimum of 3–5 years until replacement trees become productive.

Modern clones selected for their drought-tolerance characteristic were not found in our plots. However, we showed that clones not necessarily selected for the drought were more resistant than hybrids or traditional common amelonado. These clones include e.g CCN51, CCN10, PS1319 and TSH1188, accessions often selected for their high yield and disease resistance in high input and low-shade conditions, which could partially explain their drought tolerance. Numerous drought-tolerant cocoa accessions have been identified [42,60,61]. However most of the candidate clones have only been assessed in greenhouses or at very early stages of growth in plantations. There is an urgent need to assess these drought-tolerant clones in farm conditions and to introduce drought-tolerant clones in the Brazilian market of cocoa varieties. Recent ENSO-related droughts could result in ‘mass selection’ of the more drought-tolerant Amelonado at farm scale. As compared to most other crops, varietal selection by growers remains marginal in cocoa in Bahia (only 30% of the cocoa trees recorded were grafts or rooted cuttings selected as high-yielding disease-tolerant material); most cocoa found in Bahia is the semi-natural Amazonian Amelonado. A combination of ‘mass selection’ of local varieties and genetically selected drought-tolerant varieties will be necessary to limit the damage in future strong droughts.

### Trends and future of cocoa production

Recent ENSO-related drought has changed the balance of cocoa production between Brazilian states; Bahia used to produce 95% of Brazilian cocoa, but since the 1990’s, Bahian production has been declining mainly because of witches’ broom whereas production in Para state has been increasing considerably since it started in the 2000’s. In 2017 just after the drought, Para state became the most productive Brazilian state. The 2015–16 ENSO- drought reduced cocoa production in Bahia, which was already weakened by chronic fungal infection. Such changes in producing areas are common in cocoa and have been described as part of boom and bust cycles [62]. In the case of Brazil, these changes will result in a decrease in extensive traditional environmentally-friendly cocoa agroforestry systems with high species diversity (‘cabrucas’), which will be replaced by simplified systems with intensive inputs. These changes reflect a trend in world cocoa production to switch from shaded agroforests to intensively managed monocrops. However, diversified agroforestry systems remains the best strategy to increase small farmers’ resilience to severe climate events [20,63–65].

World cocoa production is negatively affected by ENSO years and positively affected by la Nina years [66]. However attempts to show clear relationships between rainfall and cocoa yield at regional scales remain inconclusive [67–70]. Only extreme ENSO events cause reduced cocoa yield in Bahia. ENSO events are often associated with yield losses in crops due to drought but also to flooding [71,72]. ENSO events are also responsible for losses in vegetation cover [73] and are a threat to tropical forests [74,75]. Forest mortality due to ENSO droughts vary from 1.4 to 89%—the higher ones being due to fire (see S1 Table). In natural forests, the effects may be limited because dead trees are rapidly replaced by regrowth and many forest systems are resilient to drought, unless they burn when it takes much longer for them to recover. In the case of crops including cocoa, tree mortality means at least three years without a cocoa crop–a serious loss of income for farmers. Crops often have low resilience to extreme climate events because they are often grown in sub-optimal regions where, for example, water could be limiting. This is the case for Barro Preto, where rainfall quantities and distribution pattern are almost at the limits for cocoa production.

Thus, we have shown, for the first time on-farm, that a severe El Niño drought reduced cocoa production by 89% for the main (April) harvests in 2016 and 2017, and killed 15% of cocoa trees. Strong droughts are not uncommon in Bahia Brazil, but an eleven-months event with two successive droughts of 136 and 131 days (separated by 25 days) was unique. It is likely that such droughts will become longer and more frequent in the tropics and thus cocoa yields in such areas will be strongly reduced. Cocoa is an example of many crops grown somewhat beyond their normal climatic range; which are sensitive to drought and whose yields might be greatly reduced in future due to changed climates with stronger and more frequent droughts. Such crops could be the ‘canaries in the coal mine’ warning of problems to come due to increased intensity and frequency of droughts [72,74], both for crops and semi-natural and natural vegetation.

## Supporting information

S1 Table Review of the studies on the effect of ENSO droughts on tropical forests. (DOCX)

S2 Table Rainfall, temperature and soil water holding capacity during ENSO 2015–16, MCCS weather station, Barro Preto, Bahia. (DOCX)

S3 Table Soil water holding capacity in 10 cocoa farms, Barro Preto. (DOCX)

S4 Table Tree mortality per cocoa farms after 2015–16 ENSO drought. (DOCX)

S5 Table Yield in kg/ha per farm following five peak harvests: before (April 2015), during (April and November 2016) and after (April and November 2017) 2015–16 ENSO drought. (DOCX)

S6 Table Harvest of ripe pods for conversion factors (pod into dry cocoa bean weight). (DOCX)

S7 Table Farm variables description. (DOCX)

S8 Table Cocoa production in Brazil since 1990 (source FAO and IBGE). (DOCX)

S9 Table List of shade trees species found in Barro Preto, Bahia, Brazil. (DOCX)
