# Supplementary material for: Climate change could threaten cocoa production: Effects of 2015-16 El Niño-related drought on cocoa agroforests in Bahia, Brazil
Source: PLoS One. 2018 Jul 10;13(7):e0200454. doi: 10.1371/journal.pone.0200454 (PMC6039034; doi:10.1371/journal.pone.0200454)
Supplement: S1 Table — (DOCX) [file pone.0200454.s001.docx]

**S1 Table.** Review of the studies on the effect of ENSO droughts on tropical forests

| Location | Site | Precipitation (monthly range) | Type of forest | Annual tree mortality (normal year) | Years of drought | Comment | References |
| --- | --- | --- | --- | --- | --- | --- | --- |
| Indonesia  (Sumatra) | Bukit  Barisan Selatan National Park | - | Lowland rainforest | 24.6% (9.8%) | 1997-98 | Mainly burnt trees | (Kinnaird and O’Brien 1998) |
| Indonesian (Borneo) | East Kalimantan | - | Lowland rainforest | 25-80% by fires | 1997-98 | 21% burnt area | (Siegert et al. 2001) |
| Indonesia (Borneo) | East Kalimantan | 2100-2500mm | Lowland rainforest | 7.9-23.1%  11.2-28.7% | 1997-98  1997-98 |  | (Nieuwstadt 2002)  (Slik 2004) |
|  |  |  |  | 23.9-46.6% | 1997-98 | 64.2-79% by fire | (van  Nieuwstadt and Sheil 2005) |
| Malaysia  (Borneo) | Lambir Hills National Park, Sarawak | 2700mm y^-1^ (167.5- 328.4) | Lowland rainforest | 4.35-6.37 (0.89%) | 1997-98 |  | (Nakagawa et al. 2000) |
| Costa Rica | La Selva Biological Station | 3962mm y^-1^ |  | 1.4-5.6% (0.8-3.7) | 1997-98 |  | (Chazdon et al., 2005) |
| Nicaragua | Autonomous  Southern Atlantic Region | 4320 mm y^-1^  Feb to April: 75–125 mm | Lowland rainforest | ~30% (2%) | 1997-98 | Include fires mortality | (Granzow-de la Cerda et al. 2012) |
| Panama | Barro Colorado Island (BCI) | 2600 mm  Dec to April: 215 mm | Semi-deciduous  tropical moist forest | 2.75-10% (1.98%)  3% (2%) | 1982-83  1982-83 |  | (Leigh et al. 1990)  (Condit et al. 1995) |
| Argentina | Nahuel  Huapi National Park | 1400 mm  year^–1^, 60% falling during May–August. | Mountain forest | 11-57% | 1998-99 |  | (Suarez and Kitzberger 2008) |
| Brazil | Tapajós National Forest  Caxiuanã  National  Forest Reserve  All Amazon | 2000 mm,  2000-2500 mm,  Dry June to Nov. | Amazonian forest | 1.91% (1.12%)  3.36% (2.54%)  2.5% (1.25%)  13% of the area | 1997–98  2000-04  2001-08  2015-16 | Artificial drought (TFE)  Artificial drought (TFE) | (Williamson et al. 2000)  (Nepstad et al. 2007)  (da Costa et al. 2010)  (Jiménez-Muñoz et al. 2016) |
|  | Linhares, Espiritu Santo | 1200mm | Atlantic forest | 4.9%  3.8% (1.4%) | 1997–98 1982–83 |  | (Rolim et al. 2005) |
|  | Barro Preto, Bahia |  | Atlantic forest and agroforest | 22% | 2015-16 |  | (This study 2017) |
| 10 countries | Mainly Amazon and Borneo |  | Lowland tropical forests | Increasing water stress increase mortality | 1982-83, 1997-98, 2005 |  | (Phillips et al. 2010) |
| Review | World |  | All types of forest |  | From 1904 to 2008 | Big trees are more affected, varying following species | (Allen et al. 2010) |
