# Supplementary material for: Climate change could threaten cocoa production: Effects of 2015-16 El Niño-related drought on cocoa agroforests in Bahia, Brazil
Source: PLoS One. 2018 Jul 10;13(7):e0200454. doi: 10.1371/journal.pone.0200454 (PMC6039034; doi:10.1371/journal.pone.0200454)
Supplement: S2 Table — (DOCX) [file pone.0200454.s002.docx]

**S2 Table.** Rainfall, temperature and soil water holding capacity during ENSO 2015-16, MCCS weather station, Barro Preto, Bahia

| **Date** | **rainfall (mm)** | **Temperature (°C)** | **PET (mm)** | **sum rainfall for the 30 preceding days** | **sum PET for the 30 preceding days** | **sum Water in soil for the 30 preceding days** |
| --- | --- | --- | --- | --- | --- | --- |
| 01/01/2015 | 0.1 | 26.5 | 4.8 | 238.6 | 140.8 | 81.0 |
| 02/01/2015 | 0.0 | 26.5 | 4.8 | 238.6 | 141.2 | 81.0 |
| 03/01/2015 | 0.0 | 26.05 | 4.7 | 238.6 | 141.5 | 81.0 |
| 04/01/2015 | 0.0 | 27.05 | 4.9 | 238.1 | 141.8 | 81.0 |
| 05/01/2015 | 9.8 | 25.1 | 4.5 | 247.9 | 141.6 | 81.0 |
| 06/01/2015 | 3.4 | 26.15 | 4.7 | 233.2 | 141.8 | 81.0 |
| 07/01/2015 | 0.0 | 26.25 | 4.7 | 232.7 | 141.9 | 81.0 |
| 08/01/2015 | 4.3 | 24.6 | 4.5 | 236 | 141.9 | 81.0 |
| 09/01/2015 | 7.0 | 25.3 | 4.6 | 230.9 | 142.5 | 81.0 |
| 10/01/2015 | 1.2 | 26.05 | 4.7 | 209.8 | 142.7 | 81.0 |
| 11/01/2015 | 0.0 | 25.45 | 4.6 | 209.8 | 142.2 | 81.0 |
| 12/01/2015 | 0.0 | 25.6 | 4.6 | 208.8 | 142.6 | 81.0 |
| 13/01/2015 | 3.1 | 25.2 | 4.6 | 209.4 | 142.1 | 81.0 |
| 14/01/2015 | 3.2 | 25.15 | 4.6 | 192.5 | 142.1 | 81.0 |
| 15/01/2015 | 6.2 | 25.65 | 4.6 | 140.5 | 142.0 | 81.0 |
| 16/01/2015 | 1.3 | 24.15 | 4.4 | 73.3 | 141.7 | 16.6 |
| 17/01/2015 | 0.0 | 28.25 | 5.1 | 62 | 142.7 | 4.3 |
| 18/01/2015 | 0.0 | 23.25 | 4.2 | 62 | 142.4 | 4.6 |
| 19/01/2015 | 2.7 | 25.7 | 4.6 | 64.7 | 142.0 | 7.7 |
| 20/01/2015 | 0.0 | 24.65 | 4.5 | 64.7 | 141.9 | 7.8 |
| 21/01/2015 | 0.0 | 25.25 | 4.6 | 64.7 | 141.9 | 7.8 |
| 22/01/2015 | 7.6 | 25.1 | 4.5 | 72.3 | 141.6 | 15.7 |
| 23/01/2015 | 23.0 | 25.75 | 4.7 | 76.7 | 141.1 | 20.6 |
| 24/01/2015 | 7.0 | 29.5 | 5.3 | 83.7 | 141.5 | 27.2 |
| 25/01/2015 | 18.6 | 25 | 4.5 | 100.8 | 141.3 | 44.5 |
| 26/01/2015 | 0.0 | 25.35 | 4.6 | 98.5 | 141.5 | 42.0 |
| 27/01/2015 | 0.2 | 26.1 | 4.7 | 98.7 | 141.7 | 42.0 |
| 28/01/2015 | 0.0 | 27.25 | 4.9 | 98.7 | 140.6 | 43.1 |
| 29/01/2015 | 5.1 | 25.65 | 4.6 | 103.8 | 139.9 | 48.9 |
| 30/01/2015 | 0.0 |  | 0.0 | 103.8 | 135.3 | 53.5 |
| 31/01/2015 | 0.2 | 27.05 | 4.9 | 103.9 | 135.4 | 53.5 |
| 01/02/2015 | 6.0 | 26.75 | 4.8 | 109.9 | 135.4 | 59.5 |
| 02/02/2015 | 0.0 | 25.15 | 4.6 | 109.9 | 135.2 | 59.7 |
| 03/02/2015 | 0.0 | 25.75 | 4.7 | 109.9 | 135.0 | 59.9 |
| 04/02/2015 | 0.0 | 26.25 | 4.7 | 100.1 | 135.2 | 49.9 |
| 05/02/2015 | 4.6 | 26.3 | 4.8 | 101.3 | 135.2 | 51.1 |
| 06/02/2015 | 36.4 | 26.2 | 4.7 | 137.7 | 135.2 | 81.0 |
| 07/02/2015 | 7.5 | 27.05 | 4.9 | 140.9 | 135.7 | 81.0 |
| 08/02/2015 | 3.1 | 32.85 | 5.9 | 137 | 137.0 | 81.0 |
| 09/02/2015 | 0.0 | 27.5 | 5.0 | 135.8 | 137.3 | 81.0 |
| 10/02/2015 | 0.0 | 25.75 | 4.7 | 135.8 | 137.4 | 81.0 |
| 11/02/2015 | 0.0 | 27.65 | 5.0 | 135.8 | 137.7 | 81.0 |
| 12/02/2015 | 0.0 | 24.8 | 4.5 | 132.7 | 137.7 | 80.0 |
| 13/02/2015 | 0.0 | 26.1 | 4.7 | 129.5 | 137.8 | 76.7 |
| 14/02/2015 | 25.3 | 25.25 | 4.6 | 148.6 | 137.8 | 81.0 |
| 15/02/2015 | 10.0 | 26.7 | 4.8 | 157.3 | 138.2 | 81.0 |
| 16/02/2015 | 21.1 | 27 | 4.9 | 178.4 | 138.0 | 81.0 |
| 17/02/2015 | 3.1 | 28.65 | 5.2 | 181.5 | 139.0 | 81.0 |
| 18/02/2015 | 4.3 | 26.1 | 4.7 | 183.1 | 139.0 | 81.0 |
| 19/02/2015 | 3.2 | 26.75 | 4.8 | 186.3 | 139.4 | 81.0 |
| 20/02/2015 | 18.2 | 25.55 | 4.6 | 204.5 | 139.5 | 81.0 |
| 21/02/2015 | 3.6 | 25.25 | 4.6 | 200.5 | 139.5 | 81.0 |
| 22/02/2015 | 4.3 | 27 | 4.9 | 181.8 | 139.7 | 81.0 |
| 23/02/2015 | 18.2 | 25.9 | 4.7 | 193 | 139.1 | 81.0 |
| 24/02/2015 | 4.7 | 25.6 | 4.6 | 179.1 | 139.2 | 81.0 |
| 25/02/2015 | 8.8 | 26.45 | 4.8 | 187.9 | 139.4 | 81.0 |
| 26/02/2015 | 0.0 | 26.65 | 4.8 | 187.7 | 139.5 | 81.0 |
| 27/02/2015 | 0.0 | 26.95 | 4.9 | 187.7 | 139.4 | 81.0 |
| 28/02/2015 | 7.1 | 28.75 | 5.2 | 189.7 | 140.0 | 81.0 |
| 01/03/2015 | 6.5 | 27.05 | 4.9 | 196.2 | 144.9 | 81.0 |
| 02/03/2015 | 0.1 | 26.75 | 4.8 | 196.1 | 144.8 | 81.0 |
| 03/03/2015 | 2.7 | 26.7 | 4.8 | 192.8 | 144.8 | 81.0 |
| 04/03/2015 | 0.0 | 25.65 | 4.6 | 192.8 | 144.9 | 81.0 |
| 05/03/2015 | 2.9 | 26.6 | 4.8 | 195.7 | 145.1 | 81.0 |
| 06/03/2015 | 2.1 | 26.65 | 4.8 | 197.8 | 145.1 | 81.0 |
| 07/03/2015 | 0.0 | 27.15 | 4.9 | 193.2 | 145.3 | 81.0 |
| 08/03/2015 | 0.0 | 25.65 | 4.6 | 156.8 | 145.2 | 81.0 |
| 09/03/2015 | 0.0 | 25.6 | 4.6 | 149.3 | 144.9 | 81.0 |
| 10/03/2015 | 1.0 | 26.15 | 4.7 | 147.2 | 143.7 | 81.0 |
| 11/03/2015 | 2.5 | 26.5 | 4.8 | 149.7 | 143.5 | 81.0 |
| 12/03/2015 | 0.3 | 25.8 | 4.7 | 150 | 143.5 | 81.0 |
| 13/03/2015 | 3.7 | 26 | 4.7 | 153.7 | 143.2 | 81.0 |
| 14/03/2015 | 4.2 | 31.1 | 5.6 | 157.9 | 144.4 | 81.0 |
| 15/03/2015 | 0.0 | 26.2 | 4.7 | 157.9 | 144.4 | 81.0 |
| 16/03/2015 | 0.0 | 26.25 | 4.7 | 132.6 | 144.6 | 73.0 |
| 17/03/2015 | 0.0 | 26.6 | 4.8 | 122.6 | 144.6 | 63.0 |
| 18/03/2015 | 10.0 | 26.6 | 4.8 | 111.5 | 144.5 | 52.0 |
| 19/03/2015 | 0.0 | 26.6 | 4.8 | 108.4 | 144.1 | 49.3 |
| 20/03/2015 | 0.0 | 27.2 | 4.9 | 104.1 | 144.3 | 44.8 |
| 21/03/2015 | 4.1 | 26.8 | 4.8 | 105 | 144.3 | 45.7 |
| 22/03/2015 | 1.0 | 26.05 | 4.7 | 87.8 | 144.4 | 28.4 |
| 23/03/2015 | 0.0 | 27.2 | 4.9 | 84.2 | 144.8 | 24.4 |
| 24/03/2015 | 0.2 | 27.6 | 5.0 | 80.1 | 144.9 | 20.2 |
| 25/03/2015 | 0.0 | 27.95 | 5.1 | 61.9 | 145.3 | 1.6 |
| 26/03/2015 | 0.0 | 26.55 | 4.8 | 57.2 | 145.4 | 0.0 |
| 27/03/2015 | 0.0 | 24.8 | 4.5 | 48.4 | 145.1 | 0.0 |
| 28/03/2015 | 0.0 | 28.55 | 5.2 | 48.4 | 145.5 | 0.0 |
| 29/03/2015 | 0.0 | 28.25 | 5.1 | 48.4 | 145.7 | 0.0 |
| 30/03/2015 | 0.0 | 27.1 | 4.9 | 41.3 | 145.4 | 0.0 |
| 31/03/2015 | 0.0 | 27.1 | 4.9 | 34.8 | 145.4 | 0.0 |
| 01/04/2015 | 0.0 | 26.85 | 4.9 | 34.7 | 145.4 | 0.0 |
| 02/04/2015 | 0.0 | 25.15 | 4.6 | 32 | 145.2 | 0.0 |
| 03/04/2015 | 2.5 | 30.1 | 5.4 | 34.5 | 146.0 | 0.0 |
| 04/04/2015 | 0.0 | 24.1 | 4.4 | 31.6 | 145.5 | 0.0 |
| 05/04/2015 | 0.0 | 28.2 | 5.1 | 29.5 | 145.8 | 0.0 |
| 06/04/2015 | 0.0 | 28.15 | 5.1 | 29.5 | 146.0 | 0.0 |
| 07/04/2015 | 36.0 | 30.6 | 5.5 | 65.5 | 146.9 | 3.6 |
| 08/04/2015 | 0.0 | 24.4 | 4.4 | 65.5 | 146.6 | 3.9 |
| 09/04/2015 | 2.8 | 26.55 | 4.8 | 67.3 | 146.7 | 5.6 |
| 10/04/2015 | 0.0 | 26.2 | 4.7 | 64.8 | 146.7 | 3.1 |
| 11/04/2015 | 0.0 | 30.25 | 5.5 | 64.5 | 147.5 | 2.0 |
| 12/04/2015 | 0.0 | 25.55 | 4.6 | 60.8 | 147.4 | 0.0 |
| 13/04/2015 | 0.0 | 26.7 | 4.8 | 56.6 | 146.6 | 0.0 |
| 14/04/2015 | 0.0 | 25.35 | 4.6 | 56.6 | 146.4 | 0.0 |
| 15/04/2015 | 0.0 | 26.2 | 4.7 | 56.6 | 146.4 | 0.0 |
| 16/04/2015 | 0.0 | 27.05 | 4.9 | 56.6 | 146.5 | 0.0 |
| 17/04/2015 | 0.0 | 27.2 | 4.9 | 46.6 | 146.6 | 0.0 |
| 18/04/2015 | 0.0 | 27.1 | 4.9 | 46.6 | 146.7 | 0.0 |
| 19/04/2015 | 0.0 | 28.55 | 5.2 | 46.6 | 147.0 | 0.0 |
| 20/04/2015 | 0.0 | 27.4 | 5.0 | 42.5 | 147.1 | 0.0 |
| 21/04/2015 | 0.0 | 27.75 | 5.0 | 41.5 | 147.4 | 0.0 |
| 22/04/2015 | 2.0 | 27.7 | 5.0 | 43.5 | 147.5 | 0.0 |
| 23/04/2015 | 29.1 | 27.95 | 5.1 | 72.4 | 147.5 | 9.9 |
| 24/04/2015 | 3.1 | 26.75 | 4.8 | 75.5 | 147.3 | 13.2 |
| 25/04/2015 | 0.2 | 26.15 | 4.7 | 75.7 | 147.2 | 13.5 |
| 26/04/2015 | 12.4 | 26.1 | 4.7 | 88.1 | 147.5 | 25.6 |
| 27/04/2015 | 0.0 | 26.15 | 4.7 | 88.1 | 147.0 | 26.1 |
| 28/04/2015 | 0.0 | 26.5 | 4.8 | 88.1 | 146.7 | 26.4 |
| 29/04/2015 | 4.2 | 25.75 | 4.7 | 92.3 | 146.5 | 30.8 |
| 30/04/2015 | 1.2 | 24.4 | 4.4 | 93.5 | 146.0 | 32.5 |
| 01/05/2015 | 0.0 | 21.7 | 3.9 | 93.5 | 145.1 | 33.4 |
| 02/05/2015 | 0.4 | 21.75 | 3.9 | 93.9 | 144.4 | 34.5 |
| 03/05/2015 | 13.1 | 21.85 | 4.0 | 104.5 | 142.9 | 46.6 |
| 04/05/2015 | 0.0 | 25.1 | 4.5 | 104.5 | 143.1 | 46.4 |
| 05/05/2015 | 0.0 | 26.2 | 4.7 | 104.5 | 142.8 | 46.7 |
| 06/05/2015 | 0.0 | 26.5 | 4.8 | 104.5 | 142.5 | 47.0 |
| 07/05/2015 | 0.6 | 27.05 | 4.9 | 69.1 | 141.8 | 12.3 |
| 08/05/2015 | 0.0 | 25.1 | 4.5 | 69.1 | 141.9 | 12.2 |
| 09/05/2015 | 0.0 | 27.25 | 4.9 | 66.3 | 142.1 | 9.2 |
| 10/05/2015 | 0.0 | 26.05 | 4.7 | 66.3 | 142.0 | 9.3 |
| 11/05/2015 | 0.0 | 27.3 | 4.9 | 66.3 | 141.5 | 9.8 |
| 12/05/2015 | 0.0 | 25.9 | 4.7 | 66.3 | 141.6 | 9.7 |
| 13/05/2015 | 0.0 | 26.55 | 4.8 | 66.3 | 141.6 | 9.7 |
| 14/05/2015 | 1.5 | 24.05 | 4.4 | 67.8 | 141.3 | 11.5 |
| 15/05/2015 | 0.0 | 25.05 | 4.5 | 67.8 | 141.1 | 11.7 |
| 16/05/2015 | 7.1 | 22.95 | 4.2 | 74.9 | 140.4 | 19.5 |
| 17/05/2015 | 0.0 | 25.8 | 4.7 | 74.9 | 140.1 | 19.8 |
| 18/05/2015 | 0.0 | 25.55 | 4.6 | 74.9 | 139.8 | 20.1 |
| 19/05/2015 | 3.9 | 25.6 | 4.6 | 78.8 | 139.3 | 24.5 |
| 20/05/2015 | 24.0 | 23.65 | 4.3 | 102.8 | 138.6 | 49.2 |
| 21/05/2015 | 2.8 | 23.7 | 4.3 | 105.6 | 137.9 | 52.7 |
| 22/05/2015 | 4.1 | 24.45 | 4.4 | 107.7 | 137.3 | 55.4 |
| 23/05/2015 | 6.2 | 21.7 | 3.9 | 84.8 | 136.2 | 33.6 |
| 24/05/2015 | 1.0 | 24.15 | 4.4 | 82.7 | 135.7 | 32.0 |
| 25/05/2015 | 0.0 | 23.65 | 4.3 | 82.5 | 135.2 | 32.3 |
| 26/05/2015 | 4.2 | 24.05 | 4.4 | 74.3 | 134.9 | 24.4 |
| 27/05/2015 | 0.7 | 25.1 | 4.5 | 75 | 134.7 | 25.3 |
| 28/05/2015 | 0.0 | 25.25 | 4.6 | 75 | 134.5 | 25.5 |
| 29/05/2015 | 0.0 | 24.15 | 4.4 | 70.8 | 134.2 | 21.6 |
| 30/05/2015 | 3.6 | 23.25 | 4.2 | 73.2 | 134.0 | 24.2 |
| 31/05/2015 | 1.1 | 25.65 | 4.6 | 74.3 | 134.7 | 24.6 |
| 01/06/2015 | 0.5 | 23.15 | 4.2 | 74.4 | 134.9 | 24.5 |
| 02/06/2015 | 0.0 | 23.7 | 4.3 | 61.3 | 135.3 | 11.0 |
| 03/06/2015 | 10.1 | 24.05 | 4.4 | 71.4 | 135.1 | 21.3 |
| 04/06/2015 | 3.3 | 27.05 | 4.9 | 74.7 | 135.2 | 24.5 |
| 05/06/2015 | 47.0 | 23.7 | 4.3 | 121.7 | 134.7 | 72.0 |
| 06/06/2015 | 57.6 | 23.05 | 4.2 | 178.7 | 134.0 | 81.0 |
| 07/06/2015 | 4.3 | 23.65 | 4.3 | 183 | 133.7 | 81.0 |
| 08/06/2015 | 10.5 | 23.65 | 4.3 | 193.5 | 133.1 | 81.0 |
| 09/06/2015 | 28.2 | 23.15 | 4.2 | 221.7 | 132.6 | 81.0 |
| 10/06/2015 | 22.2 | 24.1 | 4.4 | 243.9 | 132.0 | 81.0 |
| 11/06/2015 | 0.0 | 25.15 | 4.6 | 243.9 | 131.8 | 81.0 |
| 12/06/2015 | 0.0 | 23.6 | 4.3 | 243.9 | 131.3 | 81.0 |
| 13/06/2015 | 3.4 | 26.1 | 4.7 | 245.8 | 131.7 | 81.0 |
| 14/06/2015 | 3.7 | 21.65 | 3.9 | 249.5 | 131.1 | 81.0 |
| 15/06/2015 | 0.0 | 26.05 | 4.7 | 242.4 | 131.6 | 81.0 |
| 16/06/2015 | 1.0 | 25.1 | 4.5 | 243.4 | 131.5 | 81.0 |
| 17/06/2015 | 11.5 | 24.35 | 4.4 | 254.9 | 131.3 | 81.0 |
| 18/06/2015 | 0.1 | 24.3 | 4.4 | 251.1 | 131.0 | 81.0 |
| 19/06/2015 | 0.0 | 24.35 | 4.4 | 227.1 | 131.2 | 81.0 |
| 20/06/2015 | 0.0 | 25.15 | 4.6 | 224.3 | 131.4 | 81.0 |
| 21/06/2015 | 12.0 | 27.15 | 4.9 | 232.2 | 131.9 | 81.0 |
| 22/06/2015 | 5.1 | 22.8 | 4.1 | 231.1 | 132.1 | 81.0 |
| 23/06/2015 | 10.0 | 21.6 | 3.9 | 240.1 | 131.7 | 81.0 |
| 24/06/2015 | 50.3 | 26.75 | 4.8 | 290.4 | 132.2 | 81.0 |
| 25/06/2015 | 13.1 | 24.55 | 4.4 | 299.3 | 132.3 | 81.0 |
| 26/06/2015 | 3.1 | 23.65 | 4.3 | 301.7 | 132.1 | 81.0 |
| 27/06/2015 | 7.2 | 21.75 | 3.9 | 308.9 | 131.4 | 81.0 |
| 28/06/2015 | 51.0 | 21.6 | 3.9 | 359.9 | 131.0 | 81.0 |
| 29/06/2015 | 27.0 | 21.15 | 3.8 | 383.3 | 130.6 | 81.0 |
| 30/06/2015 | 14.5 | 23.2 | 4.2 | 396.7 | 130.1 | 81.0 |
| 01/07/2015 | 5.6 | 22.9 | 4.1 | 401.8 | 130.1 | 81.0 |
| 02/07/2015 | 1.6 | 25.1 | 4.5 | 403.4 | 130.3 | 81.0 |
| 03/07/2015 | 2.0 | 23.0 | 4.2 | 395.3 | 130.1 | 81.0 |
| 04/07/2015 | 0.0 | 26.1 | 4.7 | 392 | 130.0 | 81.0 |
| 05/07/2015 | 0.0 | 24.6 | 4.4 | 345 | 130.1 | 81.0 |
| 06/07/2015 | 8.8 | 23.4 | 4.2 | 296.2 | 130.2 | 81.0 |
| 07/07/2015 | 1.6 | 24.6 | 4.5 | 293.5 | 130.4 | 81.0 |
| 08/07/2015 | 0.0 | 24.3 | 4.4 | 283 | 130.5 | 81.0 |
| 09/07/2015 | 0.4 | 23.7 | 4.3 | 255.2 | 130.6 | 81.0 |
| 10/07/2015 | 4.0 | 25.3 | 4.6 | 237 | 130.8 | 81.0 |
| 11/07/2015 | 9.0 | 24.9 | 4.5 | 246 | 130.7 | 81.0 |
| 12/07/2015 | 11.0 | 24.8 | 4.5 | 257 | 130.9 | 81.0 |
| 13/07/2015 | 10.6 | 24.2 | 4.4 | 264.2 | 130.6 | 81.0 |
| 14/07/2015 | 9.3 | 24.1 | 4.4 | 269.8 | 131.0 | 81.0 |
| 15/07/2015 | 3.1 | 24.5 | 4.4 | 272.9 | 130.7 | 81.0 |
| 16/07/2015 | 14.2 | 22.9 | 4.1 | 286.1 | 130.3 | 81.0 |
| 17/07/2015 | 2.1 | 22.6 | 4.1 | 276.7 | 130.0 | 81.0 |
| 18/07/2015 | 7.8 | 22.8 | 4.1 | 284.4 | 129.7 | 81.0 |
| 19/07/2015 | 3.1 | 23.8 | 4.3 | 287.5 | 129.6 | 81.0 |
| 20/07/2015 | 13.6 | 22.3 | 4.0 | 301.1 | 129.1 | 81.0 |
| 21/07/2015 | 0.2 | 23.6 | 4.3 | 289.3 | 128.5 | 81.0 |
| 22/07/2015 | 8.6 | 22.5 | 4.1 | 292.8 | 128.4 | 81.0 |
| 23/07/2015 | 7.4 | 23.0 | 4.2 | 290.2 | 128.6 | 81.0 |
| 24/07/2015 | 0.3 | 23.7 | 4.3 | 240.2 | 128.1 | 81.0 |
| 25/07/2015 | 3.2 | 23.3 | 4.2 | 230.3 | 127.8 | 81.0 |
| 26/07/2015 | 0.1 | 23.4 | 4.2 | 227.3 | 127.8 | 81.0 |
| 27/07/2015 | 0.1 | 24.3 | 4.4 | 220.2 | 128.3 | 81.0 |
| 28/07/2015 | 1.6 | 24.0 | 4.3 | 170.8 | 128.7 | 81.0 |
| 29/07/2015 | 0.3 | 24.6 | 4.4 | 144.1 | 129.3 | 81.0 |
| 30/07/2015 | 3.0 | 23.1 | 4.2 | 132.6 | 129.3 | 81.0 |
| 31/07/2015 | 0.5 | 23.1 | 4.2 | 127.5 | 129.3 | 81.0 |
| 01/08/2015 | 0.0 | 23 | 4.2 | 125.9 | 128.9 | 81.0 |
| 02/08/2015 | 1.7 | 25.05 | 4.5 | 125.6 | 129.3 | 81.0 |
| 03/08/2015 | 0.0 | 22.4 | 4.1 | 125.6 | 128.6 | 81.0 |
| 04/08/2015 | 0.3 | 21.05 | 3.8 | 125.9 | 128.0 | 81.0 |
| 05/08/2015 | 0.0 | 20 | 3.6 | 117.1 | 127.4 | 74.7 |
| 06/08/2015 | 0.0 | 21.6 | 3.9 | 115.5 | 126.9 | 73.6 |
| 07/08/2015 | 0.0 | 21.35 | 3.9 | 115.5 | 126.3 | 74.2 |
| 08/08/2015 | 2.0 | 21.2 | 3.8 | 117.1 | 125.9 | 76.2 |
| 09/08/2015 | 5.6 | 19.05 | 3.4 | 118.7 | 124.8 | 78.9 |
| 10/08/2015 | 7.6 | 21.6 | 3.9 | 117.3 | 124.2 | 78.1 |
| 11/08/2015 | 3.2 | 21.25 | 3.8 | 109.5 | 123.5 | 71.0 |
| 12/08/2015 | 4.0 | 20.3 | 3.7 | 102.9 | 122.8 | 65.1 |
| 13/08/2015 | 0.5 | 21 | 3.8 | 94.1 | 122.3 | 56.8 |
| 14/08/2015 | 23.3 | 21.7 | 3.9 | 114.3 | 121.8 | 77.5 |
| 15/08/2015 | 21.6 | 22.95 | 4.2 | 121.7 | 121.8 | 81.0 |
| 16/08/2015 | 3.3 | 22.55 | 4.1 | 122.9 | 121.8 | 81.0 |
| 17/08/2015 | 21.5 | 21.6 | 3.9 | 136.6 | 121.6 | 81.0 |
| 18/08/2015 | 25.9 | 22.65 | 4.1 | 159.4 | 121.4 | 81.0 |
| 19/08/2015 | 1.4 | 22.95 | 4.2 | 147.2 | 121.5 | 81.0 |
| 20/08/2015 | 0.1 | 22.15 | 4.0 | 147.1 | 121.2 | 81.0 |
| 21/08/2015 | 0.1 | 22.55 | 4.1 | 138.6 | 121.3 | 81.0 |
| 22/08/2015 | 0.0 | 20.9 | 3.8 | 131.2 | 120.9 | 81.0 |
| 23/08/2015 | 0.0 | 24.15 | 4.4 | 130.9 | 121.0 | 81.0 |
| 24/08/2015 | 0.0 | 21.85 | 4.0 | 127.7 | 120.7 | 81.0 |
| 25/08/2015 | 0.0 | 21 | 3.8 | 127.6 | 120.3 | 81.0 |
| 26/08/2015 | 0.0 | 23.6 | 4.3 | 127.5 | 120.2 | 81.0 |
| 27/08/2015 | 0.0 | 25 | 4.5 | 125.9 | 120.3 | 81.0 |
| 28/08/2015 | 0.0 | 24.1 | 4.4 | 125.6 | 120.3 | 81.0 |
| 29/08/2015 | 3.2 | 23.95 | 4.3 | 125.8 | 120.4 | 81.0 |
| 30/08/2015 | 9.8 | 25.45 | 4.6 | 135.1 | 120.9 | 81.0 |
| 31/08/2015 | 0.0 | 22.65 | 4.1 | 135.1 | 120.8 | 81.0 |
| 01/09/2015 | 0.0 | 22.25 | 4.0 | 133.4 | 120.3 | 81.0 |
| 02/09/2015 | 3.7 | 22.55 | 4.1 | 137.1 | 120.3 | 81.0 |
| 03/09/2015 | 0.0 | 24 | 4.3 | 136.8 | 120.8 | 81.0 |
| 04/09/2015 | 0.0 | 22.15 | 4.0 | 136.8 | 121.2 | 81.0 |
| 05/09/2015 | 0.0 | 25.55 | 4.6 | 136.8 | 121.9 | 81.0 |
| 06/09/2015 | 0.0 | 24.05 | 4.4 | 136.8 | 122.4 | 81.0 |
| 07/09/2015 | 0.0 | 26.3 | 4.8 | 134.8 | 123.4 | 81.0 |
| 08/09/2015 | 0.0 | 23.65 | 4.3 | 129.2 | 124.2 | 81.0 |
| 09/09/2015 | 0.0 | 24.4 | 4.4 | 121.6 | 124.7 | 81.0 |
| 10/09/2015 | 0.0 | 23.6 | 4.3 | 118.4 | 125.1 | 78.3 |
| 11/09/2015 | 0.0 | 25.1 | 4.5 | 114.4 | 126.0 | 73.4 |
| 12/09/2015 | 0.0 | 23.7 | 4.3 | 113.9 | 126.5 | 72.4 |
| 13/09/2015 | 0.0 | 25 | 4.5 | 90.6 | 127.1 | 48.5 |
| 14/09/2015 | 0.3 | 25.15 | 4.6 | 69.3 | 127.5 | 26.8 |
| 15/09/2015 | 0.0 | 23.15 | 4.2 | 66 | 127.6 | 23.4 |
| 16/09/2015 | 0.0 | 22.25 | 4.0 | 44.5 | 127.7 | 1.8 |
| 17/09/2015 | 0.0 | 23 | 4.2 | 18.6 | 127.8 | 0.0 |
| 18/09/2015 | 0.0 | 22.85 | 4.1 | 17.2 | 127.7 | 0.0 |
| 19/09/2015 | 0.0 | 24.3 | 4.4 | 17.1 | 128.1 | 0.0 |
| 20/09/2015 | 1.6 | 25.05 | 4.5 | 18.6 | 128.6 | 0.0 |
| 21/09/2015 | 0.0 | 23.25 | 4.2 | 18.6 | 129.0 | 0.0 |
| 22/09/2015 | 0.0 | 22.5 | 4.1 | 18.6 | 128.7 | 0.0 |
| 23/09/2015 | 0.0 | 23.6 | 4.3 | 18.6 | 129.0 | 0.0 |
| 24/09/2015 | 0.0 | 24.2 | 4.4 | 18.6 | 129.6 | 0.0 |
| 25/09/2015 | 0.0 | 23.5 | 4.3 | 18.6 | 129.6 | 0.0 |
| 26/09/2015 | 0.0 | 24.5 | 4.4 | 18.6 | 129.5 | 0.0 |
| 27/09/2015 | 0.0 | 25.6 | 4.6 | 18.6 | 129.8 | 0.0 |
| 28/09/2015 | 0.0 | 27.5 | 5.0 | 15.4 | 130.4 | 0.0 |
| 29/09/2015 | 0.0 | 24.05 | 4.4 | 5.6 | 130.2 | 0.0 |
| 30/09/2015 | 0.5 | 23.8 | 4.3 | 6.1 | 130.4 | 0.0 |
| 01/10/2015 | 0.0 | 24.7 | 4.5 | 6.1 | 130.8 | 0.0 |
| 02/10/2015 | 0.0 | 26.05 | 4.7 | 2.4 | 131.4 | 0.0 |
| 03/10/2015 | 0.0 | 24.55 | 4.4 | 2.4 | 131.5 | 0.0 |
| 04/10/2015 | 0.0 | 24.1 | 4.4 | 2.4 | 131.9 | 0.0 |
| 05/10/2015 | 0.0 | 23.95 | 4.3 | 2.4 | 131.6 | 0.0 |
| 06/10/2015 | 3.1 | 23.85 | 4.3 | 5.5 | 131.6 | 0.0 |
| 07/10/2015 | 6.2 | 22.65 | 4.1 | 11.7 | 130.9 | 0.0 |
| 08/10/2015 | 0.0 | 23.6 | 4.3 | 11.7 | 130.9 | 0.0 |
| 09/10/2015 | 0.0 | 27.75 | 5.0 | 11.7 | 131.5 | 0.0 |
| 10/10/2015 | 3.2 | 26.8 | 4.8 | 14.9 | 132.1 | 0.0 |
| 11/10/2015 | 0.1 | 23.4 | 4.2 | 15 | 131.8 | 0.0 |
| 12/10/2015 | 1.1 | 21.95 | 4.0 | 16.1 | 131.5 | 0.0 |
| 13/10/2015 | 0.6 | 25.15 | 4.6 | 16.7 | 131.5 | 0.0 |
| 14/10/2015 | 0.2 | 24.7 | 4.5 | 16.6 | 131.4 | 0.0 |
| 15/10/2015 | 0.0 | 26.8 | 4.8 | 16.6 | 132.1 | 0.0 |
| 16/10/2015 | 0.0 | 24.25 | 4.4 | 16.6 | 132.4 | 0.0 |
| 17/10/2015 | 0.0 | 23.7 | 4.3 | 16.6 | 132.6 | 0.0 |
| 18/10/2015 | 0.0 | 24.55 | 4.4 | 16.6 | 132.9 | 0.0 |
| 19/10/2015 | 0.0 | 24.15 | 4.4 | 16.6 | 132.8 | 0.0 |
| 20/10/2015 | 0.0 | 26.15 | 4.7 | 15 | 133.0 | 0.0 |
| 21/10/2015 | 0.0 | 26.05 | 4.7 | 15 | 133.5 | 0.0 |
| 22/10/2015 | 0.0 | 25.6 | 4.6 | 15 | 134.1 | 0.0 |
| 23/10/2015 | 0.0 | 24.5 | 4.4 | 15 | 134.3 | 0.0 |
| 24/10/2015 | 0.0 | 24.9 | 4.5 | 15 | 134.4 | 0.0 |
| 25/10/2015 | 0.0 | 25 | 4.5 | 15 | 134.7 | 0.0 |
| 26/10/2015 | 0.7 | 25.45 | 4.6 | 15.7 | 134.8 | 0.0 |
| 27/10/2015 | 0.0 | 25.8 | 4.7 | 15.7 | 134.9 | 0.0 |
| 28/10/2015 | 0.0 | 24.55 | 4.4 | 15.7 | 134.3 | 0.0 |
| 29/10/2015 | 25.0 | 25.45 | 4.6 | 40.7 | 134.6 | 0.0 |
| 30/10/2015 | 8.0 | 25.55 | 4.6 | 48.2 | 134.9 | 0.0 |
| 31/10/2015 | 0.0 | 23.5 | 4.3 | 48.2 | 134.7 | 0.0 |
| 01/11/2015 | 0.0 | 30 | 5.4 | 48.2 | 135.4 | 0.0 |
| 02/11/2015 | 0.0 | 28.55 | 5.2 | 48.2 | 136.1 | 0.0 |
| 03/11/2015 | 0.0 | 25.5 | 4.6 | 48.2 | 136.4 | 0.0 |
| 04/11/2015 | 0.0 | 25.1 | 4.5 | 48.2 | 136.6 | 0.0 |
| 05/11/2015 | 0.0 | 23.1 | 4.2 | 45.1 | 136.5 | 0.0 |
| 06/11/2015 | 0.0 | 26.4 | 4.8 | 38.9 | 137.1 | 0.0 |
| 07/11/2015 | 0.0 | 24.95 | 4.5 | 38.9 | 137.4 | 0.0 |
| 08/11/2015 | 0.0 | 25.9 | 4.7 | 38.9 | 137.0 | 0.0 |
| 09/11/2015 | 0.0 | 24.9 | 4.5 | 35.7 | 136.7 | 0.0 |
| 10/11/2015 | 0.0 | 26.6 | 4.8 | 35.6 | 137.3 | 0.0 |
| 11/11/2015 | 0.0 | 26.2 | 4.7 | 34.5 | 138.1 | 0.0 |
| 12/11/2015 | 0.0 | 26.8 | 4.8 | 33.9 | 138.3 | 0.0 |
| 13/11/2015 | 0.0 | 24.1 | 4.4 | 33.7 | 138.2 | 0.0 |
| 14/11/2015 | 0.0 | 25.1 | 4.5 | 33.7 | 137.9 | 0.0 |
| 15/11/2015 | 0.0 | 29.05 | 5.3 | 33.7 | 138.8 | 0.0 |
| 16/11/2015 | 0.0 | 28.75 | 5.2 | 33.7 | 139.7 | 0.0 |
| 17/11/2015 | 0.0 | 28.15 | 5.1 | 33.7 | 140.4 | 0.0 |
| 18/11/2015 | 0.0 | 26.1 | 4.7 | 33.7 | 140.7 | 0.0 |
| 19/11/2015 | 0.0 | 25.8 | 4.7 | 33.7 | 140.7 | 0.0 |
| 20/11/2015 | 0.0 |  |  | 33.7 | 135.9 | 0.0 |
| 21/11/2015 | 0.0 | 23.3 | 4.2 | 33.7 | 135.5 | 0.0 |
| 22/11/2015 | 0.0 | 26.7 | 4.8 | 33.7 | 135.9 | 0.0 |
| 23/11/2015 | 0.0 | 26.55 | 4.8 | 33.7 | 136.2 | 0.0 |
| 24/11/2015 | 0.0 | 25.95 | 4.7 | 33.7 | 136.4 | 0.0 |
| 25/11/2015 | 0.0 | 27.95 | 5.1 | 33 | 136.8 | 0.0 |
| 26/11/2015 | 0.0 | 27.85 | 5.0 | 33 | 137.2 | 0.0 |
| 27/11/2015 | 7.0 | 29.5 | 5.3 | 40 | 138.1 | 0.0 |
| 28/11/2015 | 0.0 | 26.6 | 4.8 | 15 | 138.3 | 0.0 |
| 29/11/2015 | 0.0 | 27.7 | 5.0 | 7 | 138.7 | 0.0 |
| 30/11/2015 | 0.0 | 27.25 | 4.9 | 7 | 139.4 | 0.0 |
| 01/12/2015 | 0.0 | 25.5 | 4.6 | 7 | 138.6 | 0.0 |
| 02/12/2015 | 0.0 | 26.45 | 4.8 | 7 | 138.2 | 0.0 |
| 03/12/2015 | 0.0 | 25.7 | 4.6 | 7 | 138.2 | 0.0 |
| 04/12/2015 | 0.0 | 24 | 4.3 | 7 | 138.0 | 0.0 |
| 05/12/2015 | 0.0 | 27.1 | 4.9 | 7 | 138.8 | 0.0 |
| 06/12/2015 | 0.0 | 27.55 | 5.0 | 7 | 139.0 | 0.0 |
| 07/12/2015 | 0.0 | 27.5 | 5.0 | 7 | 139.4 | 0.0 |
| 08/12/2015 | 0.0 | 28.65 | 5.2 | 7 | 139.9 | 0.0 |
| 09/12/2015 | 0.0 | 26.05 | 4.7 | 7 | 140.1 | 0.0 |
| 10/12/2015 | 0.0 | 25.95 | 4.7 | 7 | 140.0 | 0.0 |
| 11/12/2015 | 0.0 | 25.05 | 4.5 | 7 | 139.8 | 0.0 |
| 12/12/2015 | 0.0 | 27 | 4.9 | 7 | 139.8 | 0.0 |
| 13/12/2015 | 0.0 | 26.3 | 4.8 | 7 | 140.2 | 0.0 |
| 14/12/2015 | 1.8 | 27 | 4.9 | 8.8 | 140.6 | 0.0 |
| 15/12/2015 | 0.0 | 26.45 | 4.8 | 8.8 | 140.1 | 0.0 |
| 16/12/2015 | 0.0 | 26.2 | 4.7 | 8.8 | 139.7 | 0.0 |
| 17/12/2015 | 0.0 | 26.5 | 4.8 | 8.8 | 139.4 | 0.0 |
| 18/12/2015 | 0.0 | 25.1 | 4.5 | 8.8 | 139.2 | 0.0 |
| 19/12/2015 | 0.0 | 25.1 | 4.5 | 8.8 | 139.0 | 0.0 |
| 20/12/2015 | 3.2 | 27.6 | 5.0 | 12 | 144.0 | 0.0 |
| 21/12/2015 | 2.2 | 25.1 | 4.5 | 14.2 | 144.4 | 0.0 |
| 22/12/2015 | 0.0 | 26.3 | 4.8 | 14.2 | 144.3 | 0.0 |
| 23/12/2015 | 0.0 | 25.4 | 4.6 | 14.2 | 144.1 | 0.0 |
| 24/12/2015 | 2.4 | 25.55 | 4.6 | 16.6 | 144.0 | 0.0 |
| 25/12/2015 | 1.0 | 27 | 4.9 | 17.6 | 143.8 | 0.0 |
| 26/12/2015 | 0.0 | 29.05 | 5.3 | 17.6 | 144.1 | 0.0 |
| 27/12/2015 | 0.0 | 28.05 | 5.1 | 10.6 | 143.8 | 0.0 |
| 28/12/2015 | 3.0 | 27.9 | 5.0 | 13.6 | 144.0 | 0.0 |
| 29/12/2015 | 1.7 | 28.1 | 5.1 | 15.3 | 144.1 | 0.0 |
| 30/12/2015 | 0.0 | 27.65 | 5.0 | 15.3 | 144.2 | 0.0 |
| 31/12/2015 | 0.0 | 26.05 | 4.7 | 15.3 | 144.3 | 0.0 |
| 01/01/2016 | 0 | 28.5 | 5.2 | 15.3 | 144.6 | 0.0 |
| 02/01/2016 | 0 | 23.05 | 4.2 | 15.3 | 144.2 | 0.0 |
| 03/01/2016 | 2.9 | 25 | 4.5 | 18.2 | 144.3 | 0.0 |
| 04/01/2016 | 7.5 | 27.1 | 4.9 | 25.7 | 144.3 | 0.0 |
| 05/01/2016 | 1.2 | 28.1 | 5.1 | 26.9 | 144.4 | 0.0 |
| 06/01/2016 | 6 | 27 | 4.9 | 32.9 | 144.4 | 0.0 |
| 07/01/2016 | 0.4 | 27.25 | 4.9 | 33.3 | 144.1 | 0.0 |
| 08/01/2016 | 4.2 | 27.65 | 5.0 | 37.5 | 144.4 | 0.0 |
| 09/01/2016 | 1.3 | 27.4 | 5.0 | 38.8 | 144.7 | 0.0 |
| 10/01/2016 | 8.3 | 27.95 | 5.1 | 47.1 | 145.2 | 0.0 |
| 11/01/2016 | 0.1 | 28.05 | 5.1 | 47.2 | 145.4 | 0.0 |
| 12/01/2016 | 0.2 | 27.6 | 5.0 | 47.4 | 145.6 | 0.0 |
| 13/01/2016 | 0 | 28.65 | 5.2 | 45.6 | 145.9 | 0.0 |
| 14/01/2016 | 1.2 | 28.5 | 5.2 | 46.8 | 146.3 | 0.0 |
| 15/01/2016 | 0 | 27.75 | 5.0 | 46.8 | 146.6 | 0.0 |
| 16/01/2016 | 3.5 | 29.25 | 5.3 | 50.3 | 147.1 | 0.0 |
| 17/01/2016 | 22.4 | 27.75 | 5.0 | 72.7 | 147.5 | 10.2 |
| 18/01/2016 | 19.1 | 25.8 | 4.7 | 91.8 | 147.7 | 29.1 |
| 19/01/2016 | 0 | 26.65 | 4.8 | 88.6 | 147.5 | 26.1 |
| 20/01/2016 | 7.9 | 26.65 | 4.8 | 94.3 | 147.8 | 31.5 |
| 21/01/2016 | 10.6 | 25.1 | 4.5 | 104.9 | 147.5 | 42.4 |
| 22/01/2016 | 42.6 | 26.9 | 4.9 | 147.5 | 147.8 | 81.0 |
| 23/01/2016 | 11.4 | 24.55 | 4.4 | 156.5 | 147.6 | 81.0 |
| 24/01/2016 | 2.1 | 27.55 | 5.0 | 157.6 | 147.7 | 81.0 |
| 25/01/2016 | 3.1 | 27.6 | 5.0 | 160.7 | 147.5 | 81.0 |
| 26/01/2016 | 0.3 |  |  | 161 | 142.4 | 81.0 |
| 27/01/2016 | 5 | 26.7 | 4.8 | 163 | 142.2 | 81.0 |
| 28/01/2016 | 1.5 | 27 | 4.9 | 162.8 | 142.0 | 81.0 |
| 29/01/2016 | 0.2 | 27.1 | 4.9 | 163 | 141.9 | 81.0 |
| 30/01/2016 | 0 |  |  | 163 | 137.2 | 81.0 |
| 31/01/2016 | 0.5 | 25.85 | 4.7 | 163.5 | 136.7 | 81.0 |
| 01/02/2016 | 3 | 26.15 | 4.7 | 166.5 | 137.3 | 81.0 |
| 02/02/2016 | 0 | 25.5 | 4.6 | 163.6 | 137.3 | 81.0 |
| 03/02/2016 | 2 | 25.25 | 4.6 | 158.1 | 137.0 | 81.0 |
| 04/02/2016 | 0.8 | 25.9 | 4.7 | 157.7 | 136.6 | 81.0 |
| 05/02/2016 | 0 | 26.15 | 4.7 | 151.7 | 136.5 | 81.0 |
| 06/02/2016 | 0.2 | 27.3 | 4.9 | 151.5 | 136.5 | 81.0 |
| 07/02/2016 | 0 | 26.2 | 4.7 | 147.3 | 136.2 | 81.0 |
| 08/02/2016 | 2.5 | 27.95 | 5.1 | 148.5 | 136.3 | 81.0 |
| 09/02/2016 | 0 | 26.1 | 4.7 | 140.2 | 136.0 | 81.0 |
| 10/02/2016 | 0 | 24.85 | 4.5 | 140.1 | 135.4 | 81.0 |
| 11/02/2016 | 0 | 26.65 | 4.8 | 139.9 | 135.2 | 81.0 |
| 12/02/2016 | 0 | 28.05 | 5.1 | 139.9 | 135.1 | 81.0 |
| 13/02/2016 | 0 | 25 | 4.5 | 138.7 | 134.5 | 81.0 |
| 14/02/2016 | 0 | 27.9 | 5.0 | 138.7 | 134.5 | 81.0 |
| 15/02/2016 | 0 | 27.2 | 4.9 | 135.2 | 134.1 | 81.0 |
| 16/02/2016 | 0 | 24.05 | 4.4 | 112.8 | 133.5 | 64.3 |
| 17/02/2016 | 0.4 | 23 | 4.2 | 94.1 | 133.0 | 46.1 |
| 18/02/2016 | 0 | 22.55 | 4.1 | 94.1 | 132.2 | 46.9 |
| 19/02/2016 | 0 | 19.85 | 3.6 | 86.2 | 131.0 | 40.2 |
| 20/02/2016 | 1.9 | 22.6 | 4.1 | 77.5 | 130.5 | 32.0 |
| 21/02/2016 | 0.5 | 26 | 4.7 | 35.4 | 130.4 | 0.0 |
| 22/02/2016 | 1.4 | 27.1 | 4.9 | 25.4 | 130.8 | 0.0 |
| 23/02/2016 | 0.5 | 27.05 | 4.9 | 23.8 | 130.7 | 0.0 |
| 24/02/2016 | 0 | 26.5 | 4.8 | 20.7 | 130.5 | 0.0 |
| 25/02/2016 | 0 | 26 | 4.7 | 20.4 | 135.2 | 0.0 |
| 26/02/2016 | 7.3 |  |  | 22.7 | 130.4 | 0.0 |
| 27/02/2016 | 0 | 26.6 | 4.8 | 21.2 | 130.3 | 0.0 |
| 28/02/2016 | 0 | 28.5 | 5.2 | 21 | 130.6 | 0.0 |
| 29/02/2016 | 0 | 26.9 | 4.9 | 21 | 135.5 | 0.0 |
| 01/03/2016 | 7.3 | 27.55 | 5.0 | 27.8 | 135.8 | 0.0 |
| 02/03/2016 | 0.1 | 25.7 | 4.6 | 24.9 | 135.7 | 0.0 |
| 03/03/2016 | 0 | 26.1 | 4.7 | 24.9 | 135.8 | 0.0 |
| 04/03/2016 | 0 | 24.8 | 4.5 | 22.9 | 135.7 | 0.0 |
| 05/03/2016 | 1.1 | 26.65 | 4.8 | 23.2 | 135.9 | 0.0 |
| 06/03/2016 | 0 | 27.45 | 5.0 | 23.2 | 136.1 | 0.0 |
| 07/03/2016 | 0.4 | 27.2 | 4.9 | 23.4 | 136.1 | 0.0 |
| 08/03/2016 | 0 | 27.5 | 5.0 | 23.4 | 136.3 | 0.0 |
| 09/03/2016 | 0.3 | 25.5 | 4.6 | 21.2 | 135.9 | 0.0 |
| 10/03/2016 | 0 | 27.15 | 4.9 | 21.2 | 136.1 | 0.0 |
| 11/03/2016 | 0 | 23.9 | 4.3 | 21.2 | 135.9 | 0.0 |
| 12/03/2016 | 0.4 | 26.55 | 4.8 | 21.6 | 135.9 | 0.0 |
| 13/03/2016 | 9.6 | 26.45 | 4.8 | 31.2 | 135.6 | 0.0 |
| 14/03/2016 | 10.5 | 25.85 | 4.7 | 41.7 | 135.7 | 0.0 |
| 15/03/2016 | 6.7 | 26.65 | 4.8 | 48.4 | 135.5 | 0.0 |
| 16/03/2016 | 6.6 | 26 | 4.7 | 55 | 135.3 | 4.7 |
| 17/03/2016 | 3.4 | 25.65 | 4.6 | 58.4 | 135.6 | 7.8 |
| 18/03/2016 | 0 | 25.95 | 4.7 | 58 | 136.1 | 6.9 |
| 19/03/2016 | 0.2 | 26.65 | 4.8 | 58.2 | 136.8 | 6.4 |
| 20/03/2016 | 0 | 26.7 | 4.8 | 58.2 | 138.1 | 5.1 |
| 21/03/2016 | 0 | 27.05 | 4.9 | 56.3 | 138.9 | 2.4 |
| 22/03/2016 | 0 | 27 | 4.9 | 55.8 | 139.1 | 1.7 |
| 23/03/2016 | 1.4 | 25.3 | 4.6 | 55.8 | 138.7 | 2.1 |
| 24/03/2016 | 0 | 27.55 | 5.0 | 55.3 | 138.8 | 1.5 |
| 25/03/2016 | 3.1 | 27 | 4.9 | 58.4 | 138.9 | 4.5 |
| 26/03/2016 | 8.9 | 26.55 | 4.8 | 67.3 | 139.0 | 13.3 |
| 27/03/2016 | 0.6 | 31.05 | 5.6 | 60.6 | 144.6 | 1.0 |
| 28/03/2016 | 0 | 26.55 | 4.8 | 60.6 | 144.6 | 1.0 |
| 29/03/2016 | 0 | 25.8 | 4.7 | 60.6 | 144.1 | 1.5 |
| 30/03/2016 | 1.9 | 25.8 | 4.7 | 62.5 | 143.9 | 3.6 |
| 31/03/2016 | 0 | 25.85 | 4.7 | 55.2 | 143.6 | 0.0 |
| 01/04/2016 | 3.4 | 26.45 | 4.8 | 58.5 | 143.8 | 0.0 |
| 02/04/2016 | 7 | 26.05 | 4.7 | 65.5 | 143.8 | 6.7 |
| 03/04/2016 | 6.9 | 25.2 | 4.6 | 72.4 | 143.8 | 13.6 |
| 04/04/2016 | 3.1 | 27.4 | 5.0 | 74.4 | 144.0 | 15.4 |
| 05/04/2016 | 0 | 27.05 | 4.9 | 74.4 | 143.9 | 15.5 |
| 06/04/2016 | 0 | 26.95 | 4.9 | 74 | 143.9 | 15.1 |
| 07/04/2016 | 0 | 25.5 | 4.6 | 74 | 143.5 | 15.5 |
| 08/04/2016 | 0.6 | 26.55 | 4.8 | 74.3 | 143.7 | 15.6 |
| 09/04/2016 | 1.5 | 24.7 | 4.5 | 75.8 | 143.2 | 17.6 |
| 10/04/2016 | 0.5 | 26 | 4.7 | 76.3 | 143.6 | 17.7 |
| 11/04/2016 | 14.4 | 25 | 4.5 | 90.3 | 143.3 | 32.0 |
| 12/04/2016 | 7 | 26.25 | 4.7 | 87.7 | 143.3 | 29.4 |
| 13/04/2016 | 0.5 | 25.5 | 4.6 | 77.7 | 143.2 | 19.5 |
| 14/04/2016 | 1.9 | 26.1 | 4.7 | 72.9 | 143.1 | 14.8 |
| 15/04/2016 | 2.7 | 24.95 | 4.5 | 69 | 143.0 | 11.0 |
| 16/04/2016 | 3.9 | 25.35 | 4.6 | 69.5 | 142.9 | 11.6 |
| 17/04/2016 | 8.6 | 28.05 | 5.1 | 78.1 | 143.3 | 19.8 |
| 18/04/2016 | 18.4 | 25.5 | 4.6 | 96.3 | 143.1 | 38.2 |
| 19/04/2016 | 1.4 | 25.25 | 4.6 | 97.7 | 142.8 | 39.9 |
| 20/04/2016 | 0.1 | 25.55 | 4.6 | 97.8 | 142.5 | 40.3 |
| 21/04/2016 | 2.3 | 26.2 | 4.7 | 100.1 | 142.4 | 42.7 |
| 22/04/2016 | 0.5 | 23.7 | 4.3 | 99.2 | 142.1 | 42.1 |
| 23/04/2016 | 2.9 | 24.4 | 4.4 | 102.1 | 141.5 | 45.6 |
| 24/04/2016 | 6.3 | 24.75 | 4.5 | 105.3 | 141.1 | 49.2 |
| 25/04/2016 | 0 | 25.05 | 4.5 | 96.4 | 140.9 | 40.5 |
| 26/04/2016 | 0 | 25.45 | 4.6 | 95.8 | 139.8 | 41.0 |
| 27/04/2016 | 0 | 25.95 | 4.7 | 95.8 | 139.7 | 41.1 |
| 28/04/2016 | 0.7 | 26.15 | 4.7 | 96.5 | 139.8 | 41.7 |
| 29/04/2016 | 1.2 | 25.05 | 4.5 | 95.8 | 139.7 | 41.1 |
| 30/04/2016 | 0 | 25.2 | 4.6 | 95.8 | 139.5 | 41.3 |
| 01/05/2016 | 11.6 | 25.5 | 4.6 | 104 | 139.4 | 49.6 |
| 02/05/2016 | 3.1 | 26.45 | 4.8 | 100.1 | 139.4 | 45.7 |
| 03/05/2016 | 0 | 25 | 4.5 | 93.2 | 139.4 | 38.8 |
| 04/05/2016 | 0 | 26.15 | 4.7 | 90.1 | 139.2 | 35.9 |
| 05/05/2016 | 0 | 25.5 | 4.6 | 90.1 | 138.9 | 36.2 |
| 06/05/2016 | 5.5 | 24.55 | 4.4 | 95.6 | 138.5 | 42.1 |
| 07/05/2016 | 10 | 20.6 | 3.7 | 105.6 | 137.6 | 53.0 |
| 08/05/2016 | 0 | 25.9 | 4.7 | 105 | 137.5 | 52.5 |
| 09/05/2016 | 0 | 23.2 | 4.2 | 103.5 | 137.2 | 51.3 |
| 10/05/2016 | 0 | 23.75 | 4.3 | 103 | 136.8 | 51.2 |
| 11/05/2016 | 0 | 25.1 | 4.5 | 88.6 | 136.8 | 36.8 |
| 12/05/2016 | 0 | 25.05 | 4.5 | 81.6 | 136.6 | 30.0 |
| 13/05/2016 | 0 | 23.65 | 4.3 | 81.1 | 136.3 | 29.8 |
| 14/05/2016 | 1.1 | 26.5 | 4.8 | 80.3 | 136.3 | 29.0 |
| 15/05/2016 | 4.5 | 25.1 | 4.5 | 82.1 | 136.3 | 30.8 |
| 16/05/2016 | 0 | 25.05 | 4.5 | 78.2 | 136.3 | 26.9 |
| 17/05/2016 | 0 | 24.35 | 4.4 | 69.6 | 135.6 | 19.0 |
| 18/05/2016 | 0 | 25.7 | 4.6 | 51.2 | 135.7 | 0.5 |
| 19/05/2016 | 0 | 25.05 | 4.5 | 49.8 | 135.6 | 0.0 |
| 20/05/2016 | 3 | 24 | 4.3 | 52.7 | 135.3 | 2.4 |
| 21/05/2016 | 3.6 | 25.4 | 4.6 | 54 | 135.2 | 3.8 |
| 22/05/2016 | 0 | 25.5 | 4.6 | 53.5 | 135.5 | 3.0 |
| 23/05/2016 | 0 | 24.55 | 4.4 | 50.6 | 135.6 | 0.0 |
| 24/05/2016 | 1.9 | 24.6 | 4.5 | 46.2 | 135.5 | 0.0 |
| 25/05/2016 | 3.8 | 18.35 | 3.3 | 50 | 134.3 | 0.7 |
| 26/05/2016 | 0 | 23.6 | 4.3 | 50 | 134.0 | 1.0 |
| 27/05/2016 | 0 | 22.45 | 4.1 | 50 | 133.3 | 1.7 |
| 28/05/2016 | 3.8 | 24.95 | 4.5 | 53.1 | 133.1 | 5.0 |
| 29/05/2016 | 1.2 | 24.05 | 4.4 | 53.1 | 132.9 | 5.2 |
| 30/05/2016 | 0 | 25 | 4.5 | 53.1 | 132.9 | 5.2 |
| 31/05/2016 | 0 | 24.55 | 4.4 | 41.5 | 132.7 | 0.0 |
| 01/06/2016 | 0 | 24.05 | 4.4 | 38.4 | 132.3 | 0.0 |
| 02/06/2016 | 0 | 27.6 | 5.0 | 38.4 | 132.8 | 0.0 |
| 03/06/2016 | 0 | 23.3 | 4.2 | 38.4 | 132.3 | 0.0 |
| 04/06/2016 | 0 | 26.5 | 4.8 | 38.4 | 132.4 | 0.0 |
| 05/06/2016 | 0 | 25.75 | 4.7 | 32.9 | 132.7 | 0.0 |
| 06/06/2016 | 0 | 25.1 | 4.5 | 22.9 | 133.5 | 0.0 |
| 07/06/2016 | 0 | 25.4 | 4.6 | 22.9 | 133.4 | 0.0 |
| 08/06/2016 | 0 | 27.3 | 4.9 | 22.9 | 134.1 | 0.0 |
| 09/06/2016 | 0 | 25.15 | 4.6 | 22.9 | 134.4 | 0.0 |
| 10/06/2016 | 0 | 24.15 | 4.4 | 22.9 | 134.2 | 0.0 |
| 11/06/2016 | 6 | 29.55 | 5.3 | 28.9 | 135.0 | 0.0 |
| 12/06/2016 | 18.8 | 24.75 | 4.5 | 47.7 | 135.2 | 0.0 |
| 13/06/2016 | 2.4 | 23.55 | 4.3 | 49 | 134.7 | 0.0 |
| 14/06/2016 | 0 | 23.1 | 4.2 | 44.5 | 134.3 | 0.0 |
| 15/06/2016 | 0 | 23.95 | 4.3 | 44.5 | 134.1 | 0.0 |
| 16/06/2016 | 0 | 21.05 | 3.8 | 44.5 | 133.5 | 0.0 |
| 17/06/2016 | 0 | 20.55 | 3.7 | 44.5 | 132.6 | 0.0 |
| 18/06/2016 | 0 | 21.8 | 3.9 | 44.5 | 132.0 | 0.0 |
| 19/06/2016 | 2.1 | 22.25 | 4.0 | 43.6 | 131.7 | 0.0 |
| 20/06/2016 | 1.2 | 23.65 | 4.3 | 41.2 | 131.4 | 0.0 |
| 21/06/2016 | 0 | 22.1 | 4.0 | 41.2 | 130.8 | 0.0 |
| 22/06/2016 | 0.9 | 22.15 | 4.0 | 42.1 | 130.3 | 0.0 |
| 23/06/2016 | 0 | 23.05 | 4.2 | 40.2 | 130.0 | 0.0 |
| 24/06/2016 | 6.1 | 26.5 | 4.8 | 42.5 | 131.5 | 0.0 |
| 25/06/2016 | 16.5 | 23.65 | 4.3 | 59 | 131.5 | 12.5 |
| 26/06/2016 | 0.5 | 22.6 | 4.1 | 59.5 | 131.6 | 12.9 |
| 27/06/2016 | 10.2 | 22.5 | 4.1 | 65.9 | 131.1 | 19.8 |
| 28/06/2016 | 14.5 | 22.55 | 4.1 | 79.2 | 130.8 | 33.4 |
| 29/06/2016 | 2.5 | 24.45 | 4.4 | 81.7 | 130.7 | 36.0 |
| 30/06/2016 | 6.2 | 21.65 | 3.9 | 87.9 | 130.2 | 42.7 |
| 01/07/2016 | 11.6 | 20.9 | 3.8 | 99.5 | 129.6 | 54.9 |
| 02/07/2016 | 4.3 | 24.6 | 4.5 | 103.8 | 129.1 | 59.7 |
| 03/07/2016 | 10.7 | 23.05 | 4.2 | 114.5 | 129.1 | 70.4 |
| 04/07/2016 | 9.4 | 24.3 | 4.4 | 123.9 | 128.7 | 80.2 |
| 05/07/2016 | 0.6 | 23.9 | 4.3 | 124.5 | 128.3 | 81.0 |
| 06/07/2016 | 0.1 | 26.2 | 4.7 | 124.6 | 128.5 | 81.0 |
| 07/07/2016 | 0.2 | 26.55 | 4.8 | 124.8 | 128.7 | 81.0 |
| 08/07/2016 | 0 | 20.95 | 3.8 | 124.8 | 127.6 | 81.0 |
| 09/07/2016 | 2.7 | 19.55 | 3.5 | 127.5 | 126.6 | 81.0 |
| 10/07/2016 | 0.1 | 24.95 | 4.5 | 127.6 | 126.7 | 81.0 |
| 11/07/2016 | 0.2 | 27 | 4.9 | 121.8 | 126.3 | 80.5 |
| 12/07/2016 | 0 | 21.7 | 3.9 | 103 | 125.7 | 62.3 |
| 13/07/2016 | 0 | 22.05 | 4.0 | 100.6 | 125.4 | 60.2 |
| 14/07/2016 | 5.4 | 22.85 | 4.1 | 106 | 125.4 | 65.6 |
| 15/07/2016 | 1.5 | 24.6 | 4.5 | 107.5 | 125.5 | 67.0 |
| 16/07/2016 | 0 | 23.5 | 4.3 | 107.5 | 125.9 | 66.6 |
| 17/07/2016 | 0 | 23.45 | 4.2 | 107.5 | 126.5 | 66.0 |
| 18/07/2016 | 0 | 23.45 | 4.2 | 107.5 | 126.8 | 65.7 |
| 19/07/2016 | 25.3 | 22.9 | 4.1 | 130.7 | 126.9 | 81.0 |
| 20/07/2016 | 10.4 | 22.6 | 4.1 | 139.9 | 126.7 | 81.0 |
| 21/07/2016 | 0.1 | 22.7 | 4.1 | 140 | 126.8 | 81.0 |
| 22/07/2016 | 6.4 | 23.95 | 4.3 | 145.5 | 127.1 | 81.0 |
| 23/07/2016 | 4.6 | 21.5 | 3.9 | 150.1 | 126.9 | 81.0 |
| 24/07/2016 | 12.5 | 19.15 | 3.5 | 156.5 | 125.5 | 81.0 |
| 25/07/2016 | 11.1 | 17.35 | 3.1 | 151.1 | 124.4 | 81.0 |
| 26/07/2016 | 0.6 | 19.8 | 3.6 | 151.2 | 123.9 | 81.0 |
| 27/07/2016 | 0.2 | 21.35 | 3.9 | 141.2 | 123.7 | 81.0 |
| 28/07/2016 | 2.1 | 21.4 | 3.9 | 128.8 | 123.5 | 81.0 |
| 29/07/2016 | 0 | 21.4 | 3.9 | 126.3 | 122.9 | 81.0 |
| 30/07/2016 | 12.1 | 25.8 | 4.7 | 132.2 | 123.7 | 81.0 |
| 31/07/2016 | 0 | 23.3 | 4.2 | 120.6 | 124.1 | 81.0 |
| 01/08/2016 | 8.9 | 19.55 | 3.5 | 125.2 | 123.2 | 81.0 |
| 02/08/2016 | 21.4 | 22.6 | 4.1 | 135.9 | 123.1 | 81.0 |
| 03/08/2016 | 3.4 | 23.1 | 4.2 | 129.9 | 122.9 | 81.0 |
| 04/08/2016 | 2.5 | 24.55 | 4.4 | 131.8 | 123.0 | 81.0 |
| 05/08/2016 | 13.1 | 17.85 | 3.2 | 144.8 | 121.5 | 81.0 |
| 06/08/2016 | 12.8 | 20.6 | 3.7 | 157.4 | 120.4 | 81.0 |
| 07/08/2016 | 0 | 25.1 | 4.5 | 157.4 | 121.2 | 81.0 |
| 08/08/2016 | 0 | 22.9 | 4.1 | 154.7 | 121.8 | 81.0 |
| 09/08/2016 | 0 | 22.15 | 4.0 | 154.6 | 121.3 | 81.0 |
| 10/08/2016 | 0 | 22.6 | 4.1 | 154.4 | 120.5 | 81.0 |
| 11/08/2016 | 0 | 24 | 4.3 | 154.4 | 120.9 | 81.0 |
| 12/08/2016 | 0 | 23.5 | 4.3 | 154.4 | 121.1 | 81.0 |
| 13/08/2016 | 0 | 21.5 | 3.9 | 149 | 120.9 | 81.0 |
| 14/08/2016 | 0 | 23.4 | 4.2 | 147.5 | 120.7 | 81.0 |
| 15/08/2016 | 0 | 22.7 | 4.1 | 147.5 | 120.5 | 81.0 |
| 16/08/2016 | 0 | 22.6 | 4.1 | 147.5 | 120.4 | 81.0 |
| 17/08/2016 | 0 | 25 | 4.5 | 147.5 | 120.7 | 81.0 |
| 18/08/2016 | 0 | 24.05 | 4.4 | 122.2 | 120.9 | 81.0 |
| 19/08/2016 | 2.6 | 23.55 | 4.3 | 114.4 | 121.0 | 78.4 |
| 20/08/2016 | 0 | 23.15 | 4.2 | 114.3 | 121.1 | 78.2 |
| 21/08/2016 | 0.1 | 26.55 | 4.8 | 108 | 121.6 | 71.4 |
| 22/08/2016 | 0.1 | 23.5 | 4.3 | 103.5 | 122.0 | 66.5 |
| 23/08/2016 | 9.7 | 25.05 | 4.5 | 100.7 | 123.0 | 62.7 |
| 24/08/2016 | 1 | 21.5 | 3.9 | 90.6 | 123.8 | 51.8 |
| 25/08/2016 | 0 | 22.05 | 4.0 | 90 | 124.2 | 50.8 |
| 26/08/2016 | 6.2 | 21.15 | 3.8 | 96 | 124.1 | 56.9 |
| 27/08/2016 | 3.4 | 19.4 | 3.5 | 97.3 | 123.8 | 58.5 |
| 28/08/2016 | 19.6 | 22.7 | 4.1 | 116.9 | 124.0 | 77.9 |
| 29/08/2016 | 26.1 | 24.85 | 4.5 | 130.9 | 123.8 | 81.0 |
| 30/08/2016 | 3.9 | 25.4 | 4.6 | 134.8 | 124.2 | 81.0 |
| 31/08/2016 | 1.4 | 24.25 | 4.4 | 127.3 | 125.1 | 81.0 |
| 01/09/2016 | 5.8 | 22.55 | 4.1 | 111.7 | 125.1 | 71.6 |
| 02/09/2016 | 0.0 | 22.5 | 4.1 | 108.3 | 125.0 | 68.3 |
| 03/09/2016 | 4.8 | 22.35 | 4.0 | 110.6 | 124.6 | 71.0 |
| 04/09/2016 | 6.0 | 22.95 | 4.2 | 103.5 | 125.5 | 63.0 |
| 05/09/2016 | 1.0 | 23.55 | 4.3 | 91.7 | 126.0 | 50.7 |
| 06/09/2016 | 0.0 | 24.65 | 4.5 | 91.7 | 125.9 | 50.8 |
| 07/09/2016 | 0.6 | 26.25 | 4.7 | 92.3 | 126.5 | 50.8 |
| 08/09/2016 | 0.0 | 22.4 | 4.1 | 92.3 | 126.6 | 50.7 |
| 09/09/2016 | 18.2 | 24.1 | 4.4 | 110.5 | 126.9 | 68.6 |
| 10/09/2016 | 0.4 | 28.25 | 5.1 | 110.9 | 127.6 | 68.3 |
| 11/09/2016 | 1.5 | 22.5 | 4.1 | 112.4 | 127.4 | 70.0 |
| 12/09/2016 | 0.8 | 22.5 | 4.1 | 113.2 | 127.6 | 70.6 |
| 13/09/2016 | 0.0 | 22.95 | 4.2 | 113.2 | 127.5 | 70.7 |
| 14/09/2016 | 0.0 | 20.9 | 3.8 | 113.2 | 127.2 | 71.0 |
| 15/09/2016 | 0.0 | 22.05 | 4.0 | 113.2 | 127.1 | 71.1 |
| 16/09/2016 | 0.0 | 21.05 | 3.8 | 113.2 | 126.4 | 71.8 |
| 17/09/2016 | 3.4 | 23.25 | 4.2 | 116.6 | 126.3 | 75.3 |
| 18/09/2016 | 0.0 | 23.1 | 4.2 | 114 | 126.2 | 72.8 |
| 19/09/2016 | 0.0 | 24.15 | 4.4 | 114 | 126.4 | 72.6 |
| 20/09/2016 | 0.0 | 23.45 | 4.2 | 113.9 | 125.8 | 73.1 |
| 21/09/2016 | 1.2 | 24.2 | 4.4 | 115 | 125.9 | 74.1 |
| 22/09/2016 | 0.1 | 23.1 | 4.2 | 105.4 | 125.6 | 64.8 |
| 23/09/2016 | 0.0 | 20.05 | 3.6 | 104.4 | 125.3 | 64.1 |
| 24/09/2016 | 0.7 | 25.35 | 4.6 | 105.1 | 125.9 | 64.2 |
| 25/09/2016 | 3.3 | 22.8 | 4.1 | 102.2 | 126.2 | 61.0 |
| 26/09/2016 | 4.2 | 24.95 | 4.5 | 103 | 127.2 | 60.8 |
| 27/09/2016 | 11.9 | 23.55 | 4.3 | 95.3 | 127.4 | 52.9 |
| 28/09/2016 | 3.5 | 24.85 | 4.5 | 72.7 | 127.4 | 30.3 |
| 29/09/2016 | 0.0 | 25.4 | 4.6 | 68.8 | 127.4 | 26.4 |
| 30/09/2016 | 0.0 | 24.25 | 4.4 | 67.4 | 127.4 | 25.0 |
| 01/10/2016 | 22.7 | 26.1 | 4.7 | 84.3 | 128.0 | 41.3 |
| 02/10/2016 | 19.0 | 25.6 | 4.6 | 103.3 | 128.6 | 59.7 |
| 03/10/2016 | 21.8 | 23.65 | 4.3 | 120.3 | 128.8 | 76.5 |
| 04/10/2016 | 5.7 | 22.15 | 4.0 | 120 | 128.7 | 76.3 |
| 05/10/2016 | 0.2 | 25.55 | 4.6 | 119.2 | 129.0 | 75.2 |
| 06/10/2016 | 2.2 | 25.05 | 4.5 | 121.4 | 129.1 | 77.3 |
| 07/10/2016 | 2.8 | 20.65 | 3.7 | 123.6 | 128.1 | 80.5 |
| 08/10/2016 | 0.0 | 19.2 | 3.5 | 123.6 | 127.5 | 81.0 |
| 09/10/2016 | 3.5 | 28.65 | 5.2 | 108.9 | 128.3 | 65.6 |
| 10/10/2016 | 0.9 | 25.65 | 4.6 | 109.4 | 127.9 | 66.5 |
| 11/10/2016 | 29.0 | 20.6 | 3.7 | 136.9 | 127.5 | 81.0 |
| 12/10/2016 | 5.5 | 27.6 | 5.0 | 141.6 | 128.4 | 81.0 |
| 13/10/2016 | 27.1 | 23.6 | 4.3 | 168.7 | 128.6 | 81.0 |
| 14/10/2016 | 0.3 | 24.95 | 4.5 | 169 | 129.3 | 81.0 |
| 15/10/2016 | 0.0 | 24.4 | 4.4 | 169 | 129.7 | 81.0 |
| 16/10/2016 | 0.9 | 25.55 | 4.6 | 169.9 | 130.5 | 81.0 |
| 17/10/2016 | 5.8 | 24.05 | 4.4 | 172.3 | 130.7 | 81.0 |
| 18/10/2016 | 0.5 | 24 | 4.3 | 172.8 | 130.8 | 81.0 |
| 19/10/2016 | 0.0 | 24.95 | 4.5 | 172.8 | 131.0 | 81.0 |
| 20/10/2016 | 0.5 | 23.7 | 4.3 | 173.3 | 131.0 | 81.0 |
| 21/10/2016 | 0.0 | 23.6 | 4.3 | 172.1 | 130.9 | 81.0 |
| 22/10/2016 | 10.6 | 24.45 | 4.4 | 182.6 | 131.2 | 81.0 |
| 23/10/2016 | 7.1 | 24.3 | 4.4 | 189.7 | 131.9 | 81.0 |
| 24/10/2016 | 0.0 | 18.05 | 3.3 | 189 | 130.6 | 81.0 |
| 25/10/2016 | 0.0 | 16.65 | 3.0 | 185.7 | 129.5 | 81.0 |
| 26/10/2016 | 0.0 | 23.1 | 4.2 | 181.5 | 129.2 | 81.0 |
| 27/10/2016 | 0.0 | 23.65 | 4.3 | 169.6 | 129.2 | 81.0 |
| 28/10/2016 | 4.0 | 24.85 | 4.5 | 170.1 | 129.2 | 81.0 |
| 29/10/2016 | 0.0 | 26.75 | 4.8 | 170.1 | 129.4 | 81.0 |
| 30/10/2016 | 9.0 | 25.1 | 4.5 | 179.1 | 129.6 | 81.0 |
| 31/10/2016 | 0.0 | 25.5 | 4.6 | 156.4 | 129.5 | 81.0 |
| 01/11/2016 | 10.0 | 25.05 | 4.5 | 147.4 | 129.4 | 81.0 |
| 02/11/2016 | 0.0 | 26.15 | 4.7 | 125.6 | 129.8 | 80.8 |
| 03/11/2016 | 7.1 | 25.7 | 4.6 | 127 | 130.5 | 81.0 |
| 04/11/2016 | 5.3 | 25.55 | 4.6 | 132.1 | 130.5 | 81.0 |
| 05/11/2016 | 6.5 | 20.7 | 3.7 | 136.4 | 129.7 | 81.0 |
| 06/11/2016 | 16.3 | 23.15 | 4.2 | 149.9 | 130.1 | 81.0 |
| 07/11/2016 | 16.0 | 25.3 | 4.6 | 165.9 | 131.2 | 81.0 |
| 08/11/2016 | 14.6 | 24.2 | 4.4 | 177 | 130.4 | 81.0 |
| 09/11/2016 | 0.7 | 25.1 | 4.5 | 176.8 | 130.3 | 81.0 |
| 10/11/2016 | 0.6 | 24.7 | 4.5 | 148.4 | 131.1 | 81.0 |
| 11/11/2016 | 10.2 | 25 | 4.5 | 153.1 | 130.6 | 81.0 |
| 12/11/2016 | 4.1 | 26.15 | 4.7 | 130.1 | 131.1 | 81.0 |
| 13/11/2016 | 18.4 | 25.3 | 4.6 | 148.2 | 131.1 | 81.0 |
| 14/11/2016 | 15.4 | 23.4 | 4.2 | 163.6 | 130.9 | 81.0 |
| 15/11/2016 | 24.2 | 24.65 | 4.5 | 186.9 | 130.8 | 81.0 |
| 16/11/2016 | 6.3 | 25.4 | 4.6 | 187.4 | 131.0 | 81.0 |
| 17/11/2016 | 0.1 | 26.85 | 4.9 | 187 | 131.5 | 81.0 |
| 18/11/2016 | 0.0 | 22.1 | 4.0 | 187 | 131.0 | 81.0 |
| 19/11/2016 | 1.6 | 21.1 | 3.8 | 188.1 | 130.6 | 81.0 |
| 20/11/2016 | 0.0 | 23.6 | 4.3 | 188.1 | 130.6 | 81.0 |
| 21/11/2016 | 1.5 | 23.9 | 4.3 | 179 | 130.5 | 81.0 |
| 22/11/2016 | 0.0 | 26.55 | 4.8 | 171.9 | 130.9 | 81.0 |
| 23/11/2016 | 0.0 | 25.45 | 4.6 | 171.9 | 132.2 | 81.0 |
| 24/11/2016 | 0.0 | 25.9 | 4.7 | 171.9 | 133.9 | 81.0 |
| 25/11/2016 | 0.0 | 25.55 | 4.6 | 171.9 | 134.3 | 81.0 |
| 26/11/2016 | 0.0 | 25.5 | 4.6 | 171.9 | 134.6 | 81.0 |
| 27/11/2016 | 0.0 | 25 | 4.5 | 167.9 | 134.7 | 81.0 |
| 28/11/2016 | 0.0 | 25.9 | 4.7 | 167.9 | 134.5 | 81.0 |
| 29/11/2016 | 0.0 | 26.15 | 4.7 | 158.9 | 134.7 | 81.0 |
| 30/11/2016 | 0.0 | 25.25 | 4.6 | 158.9 | 134.7 | 81.0 |
| 01/12/2016 | 0.0 | 25.75 | 4.7 | 148.9 | 134.8 | 81.0 |
| 02/12/2016 | 0.0 | 26.2 | 4.7 | 148.9 | 134.8 | 81.0 |
| 03/12/2016 | 1.3 | 26.7 | 4.8 | 143.1 | 135.0 | 81.0 |
| 04/12/2016 | 5.9 | 26.7 | 4.8 | 143.7 | 135.2 | 81.0 |
| 05/12/2016 | 0.2 | 26.9 | 4.9 | 137.4 | 136.3 | 81.0 |
| 06/12/2016 | 0.0 | 26.9 | 4.9 | 121.1 | 137.0 | 69.1 |
| 07/12/2016 | 0.0 | 26.95 | 4.9 | 105.1 | 137.3 | 52.8 |
| 08/12/2016 | 0.0 | 26.45 | 4.8 | 90.5 | 137.7 | 37.8 |
| 09/12/2016 | 0.2 | 25.55 | 4.6 | 90 | 137.8 | 37.2 |
| 10/12/2016 | 0.0 | 24.55 | 4.4 | 89.4 | 137.8 | 36.6 |
| 11/12/2016 | 0.0 | 24.65 | 4.5 | 79.2 | 137.7 | 26.5 |
| 12/12/2016 | 0.1 | 26.5 | 4.8 | 75.2 | 137.8 | 22.4 |
| 13/12/2016 | 0.0 | 27.05 | 4.9 | 56.8 | 138.1 | 3.7 |
| 14/12/2016 | 0.0 | 25.15 | 4.6 | 41.4 | 138.4 | 0.0 |
| 15/12/2016 | 0.0 | 25.55 | 4.6 | 17.2 | 138.5 | 0.0 |
| 16/12/2016 | 0.0 | 25.1 | 4.5 | 10.9 | 138.5 | 0.0 |
| 17/12/2016 | 15.5 | 24.4 | 4.4 | 26.3 | 138.1 | 0.0 |
| 18/12/2016 | 23.2 | 25.4 | 4.6 | 49.5 | 138.6 | 0.0 |
| 19/12/2016 | 3.5 | 25.5 | 4.6 | 51.4 | 139.4 | 0.0 |
| 20/12/2016 | 3.4 | 23.85 | 4.3 | 54.8 | 139.5 | 0.3 |
| 21/12/2016 | 0.5 | 25.65 | 4.6 | 53.8 | 139.8 | 0.0 |
| 22/12/2016 | 1.2 | 26.6 | 4.8 | 55 | 139.8 | 0.2 |
| 23/12/2016 | 3.5 | 25 | 4.5 | 58.5 | 139.7 | 3.8 |
| 24/12/2016 | 2.1 | 26.5 | 4.8 | 60.6 | 139.8 | 5.8 |
| 25/12/2016 | 0.0 | 27.5 | 5.0 | 60.6 | 140.2 | 5.4 |
| 26/12/2016 | 0.0 | 28 | 5.1 | 60.6 | 140.6 | 5.0 |
| 27/12/2016 | 1.0 | 26.05 | 4.7 | 61.6 | 140.8 | 5.8 |
| 28/12/2016 | 0.0 | 24.55 | 4.4 | 61.6 | 140.6 | 6.0 |
| 29/12/2016 | 0.0 | 27.25 | 4.9 | 61.6 | 140.8 | 5.8 |
| 30/12/2016 | 2.2 | 25.5 | 4.6 | 63.8 | 140.8 | 8.0 |
| 31/12/2016 | 0.0 | 25.15 | 4.6 | 63.8 | 140.7 | 8.1 |
| 01/01/2017 | 0.2 | 25.5 | 4.6 | 64 | 140.6 | 8.4 |
| 02/01/2017 | 0.0 | 25.5 | 4.6 | 62.7 | 140.4 | 7.3 |
| 03/01/2017 | 0.0 | 25.55 | 4.6 | 56.8 | 140.2 | 1.6 |
| 04/01/2017 | 0.0 | 25.1 | 4.5 | 56.6 | 139.9 | 1.7 |
| 05/01/2017 | 0.0 | 23.75 | 4.3 | 56.6 | 139.3 | 2.3 |
| 06/01/2017 | 0.0 | 24.55 | 4.4 | 56.6 | 138.8 | 2.8 |
| 07/01/2017 | 10.6 | 24.05 | 4.4 | 67.2 | 138.4 | 13.8 |
| 08/01/2017 | 0.4 | 25.05 | 4.5 | 67.4 | 138.3 | 14.1 |
| 09/01/2017 | 0.0 | 26.1 | 4.7 | 67.4 | 138.6 | 13.8 |
| 10/01/2017 | 0.0 | 26 | 4.7 | 67.4 | 138.8 | 13.6 |
| 11/01/2017 | 0.1 | 25.7 | 4.6 | 67.4 | 138.7 | 13.7 |
| 12/01/2017 | 0.0 | 25.05 | 4.5 | 67.4 | 138.3 | 14.1 |
| 13/01/2017 | 2.5 | 24.6 | 4.5 | 69.9 | 138.2 | 16.7 |
| 14/01/2017 | 0.0 | 25.55 | 4.6 | 69.9 | 138.2 | 16.7 |
| 15/01/2017 | 0.6 | 25.25 | 4.6 | 70.5 | 138.3 | 17.2 |
| 16/01/2017 | 1.7 | 25.05 | 4.5 | 56.7 | 138.4 | 3.3 |
| 17/01/2017 | 0.0 | 25.55 | 4.6 | 33.5 | 138.4 | 0.0 |
| 18/01/2017 | 0.6 | 24.4 | 4.4 | 30.6 | 138.2 | 0.0 |
| 19/01/2017 | 0.0 | 22.05 | 4.0 | 27.2 | 137.9 | 0.0 |
| 20/01/2017 | 0.0 | 25.55 | 4.6 | 26.7 | 137.9 | 0.0 |
| 21/01/2017 | 0.0 | 24.85 | 4.5 | 25.5 | 137.6 | 0.0 |
| 22/01/2017 | 1.3 | 26.3 | 4.8 | 23.3 | 137.8 | 0.0 |
| 23/01/2017 | 0.0 | 26.1 | 4.7 | 21.2 | 137.7 | 0.0 |
| 24/01/2017 | 1.9 | 25.6 | 4.6 | 23.1 | 137.4 | 0.0 |
| 25/01/2017 | 0.0 | 25.5 | 4.6 | 23.1 | 136.9 | 0.0 |
| 26/01/2017 | 1.0 | 25.95 | 4.7 | 23.1 | 136.9 | 0.0 |
| 27/01/2017 | 7.1 | 25.9 | 4.7 | 30.2 | 137.1 | 0.0 |
| 28/01/2017 | 12.1 | 26 | 4.6 | 42.3 | 136.8 | 0.0 |
| 29/01/2017 | 0.0 | 25.55 | 4.6 | 40.1 | 136.8 | 0.0 |
| 30/01/2017 | 0.0 | 25.65 | 4.6 | 40.1 | 136.9 | 0.0 |
| 31/01/2017 | 0.0 | 24.9 | 4.5 | 39.9 | 136.8 | 0.0 |
| 01/02/2017 | 0.0 | 25.9 | 4.7 | 39.9 | 136.9 | 0.0 |
| 02/02/2017 | 1.2 | 26.35 | 4.8 | 41.1 | 137.0 | 0.0 |
| 03/02/2017 | 5.5 | 25 | 4.5 | 46.6 | 137.0 | 0.0 |
| 04/02/2017 | 0.0 | 25.65 | 4.6 | 46.6 | 137.4 | 0.0 |
| 05/02/2017 | 17.7 | 25.9 | 4.7 | 64.3 | 137.6 | 11.7 |
| 06/02/2017 | 1.7 | 26.1 | 4.7 | 55.4 | 138.0 | 2.4 |
| 07/02/2017 | 3.8 | 24.15 | 4.4 | 58.8 | 137.8 | 6.0 |
| 08/02/2017 | 59.1 | 26.45 | 4.8 | 117.9 | 137.9 | 65.0 |
| 09/02/2017 | 28.0 | 26.25 | 4.7 | 145.9 | 137.9 | 81.0 |
| 10/02/2017 | 1.2 | 24.5 | 4.4 | 147 | 137.7 | 81.0 |
| 11/02/2017 | 0.0 | 25.3 | 4.6 | 147 | 137.8 | 81.0 |
| 12/02/2017 | 5.6 | 25.15 | 4.6 | 150.1 | 137.9 | 81.0 |
| 13/02/2017 | 2.2 | 25.5 | 4.6 | 152.3 | 137.8 | 81.0 |
| 14/02/2017 | 5.2 | 26.15 | 4.7 | 156.9 | 138.0 | 81.0 |
| 15/02/2017 | 16.2 | 21.15 | 3.8 | 171.4 | 137.3 | 81.0 |
| 16/02/2017 | 0.5 | 24.05 | 4.4 | 171.9 | 137.0 | 81.0 |
| 17/02/2017 | 0.0 | 26 | 4.7 | 171.3 | 137.3 | 81.0 |
| 18/02/2017 | 3.8 | 24.9 | 4.5 | 175.1 | 137.8 | 81.0 |
| 19/02/2017 | 11.8 | 24.65 | 4.5 | 186.9 | 137.7 | 81.0 |
| 20/02/2017 | 9.0 | 24.95 | 4.5 | 195.9 | 137.7 | 81.0 |
| 21/02/2017 | 12.0 | 25.8 | 4.7 | 206.6 | 137.6 | 81.0 |
| 22/02/2017 | 5.3 | 25.05 | 4.5 | 211.9 | 137.4 | 81.0 |
| 23/02/2017 | 1.0 | 26.05 | 4.7 | 211 | 137.5 | 81.0 |
| 24/02/2017 | 0.0 | 25.5 | 4.6 | 211 | 137.5 | 81.0 |
| 25/02/2017 | 0.0 | 24.65 | 4.5 | 210 | 137.3 | 81.0 |
| 26/02/2017 | 0.5 | 26.15 | 4.7 | 203.4 | 137.3 | 81.0 |
| 27/02/2017 | 4.2 | 25 | 4.5 | 195.5 | 137.2 | 81.0 |
| 28/02/2017 | 9.8 | 25 | 4.5 | 205.3 | 137.1 | 81.0 |
| 01/03/2017 | 0.7 | 24.6 | 4.5 | 206.0 | 136.9 | 81.0 |
| 02/03/2017 | 4.1 | 25.5 | 4.6 | 210.1 | 137.0 | 81.0 |
| 03/03/2017 | 1.3 | 25.65 | 4.6 | 211.4 | 137.0 | 81.0 |
| 04/03/2017 | 0.0 | 25.15 | 4.6 | 210.2 | 136.8 | 81.0 |
| 05/03/2017 | 0.0 | 26.4 | 4.8 | 204.7 | 137.0 | 81.0 |
| 06/03/2017 | 0.0 | 25.2 | 4.6 | 204.7 | 136.9 | 81.0 |
| 07/03/2017 | 0.0 | 25.5 | 4.6 | 187.0 | 136.9 | 81.0 |
| 08/03/2017 | 0.0 | 25.05 | 4.5 | 185.3 | 136.7 | 81.0 |
| 09/03/2017 | 8.9 | 23.55 | 4.3 | 190.4 | 136.6 | 81.0 |
| 10/03/2017 | 10.0 | 24.6 | 4.5 | 141.3 | 136.2 | 81.0 |
| 11/03/2017 | 3.5 | 24.65 | 4.5 | 116.8 | 135.9 | 65.9 |
| 12/03/2017 | 6.8 | 25.6 | 4.6 | 122.4 | 136.1 | 71.3 |
| 13/03/2017 | 3.3 | 24.6 | 4.5 | 125.7 | 136.0 | 74.7 |
| 14/03/2017 | 0.4 | 25 | 4.5 | 120.5 | 136.0 | 69.5 |
| 15/03/2017 | 4.2 | 26.45 | 4.8 | 122.5 | 136.2 | 71.3 |
| 16/03/2017 | 9.1 | 23.95 | 4.3 | 126.4 | 135.8 | 75.6 |
| 17/03/2017 | 8.2 | 25.7 | 4.6 | 118.4 | 136.6 | 66.8 |
| 18/03/2017 | 0.0 | 25.5 | 4.6 | 117.9 | 136.8 | 66.1 |
| 19/03/2017 | 0.0 | 26.05 | 4.7 | 117.9 | 136.9 | 66.0 |
| 20/03/2017 | 0.0 | 24.1 | 4.4 | 114.1 | 136.7 | 62.4 |
| 21/03/2017 | 0.5 | 25.6 | 4.6 | 102.8 | 136.9 | 50.9 |
| 22/03/2017 | 0.0 | 26.15 | 4.7 | 93.8 | 137.1 | 41.7 |
| 23/03/2017 | 0.0 | 25.1 | 4.5 | 81.8 | 137.0 | 29.8 |
| 24/03/2017 | 0.0 | 24.95 | 4.5 | 76.5 | 137.0 | 24.5 |
| 25/03/2017 | 0.0 | 29.65 | 5.4 | 75.5 | 137.6 | 22.9 |
| 26/03/2017 | 0.0 | 27 | 4.9 | 75.5 | 137.9 | 22.6 |
| 27/03/2017 | 0.0 | 26.2 | 4.7 | 75.5 | 138.2 | 22.3 |
| 28/03/2017 | 47.0 | 26.5 | 4.8 | 122.0 | 138.2 | 68.8 |
| 29/03/2017 | 3.8 | 23.9 | 4.3 | 121.6 | 138.0 | 68.6 |
| 30/03/2017 | 0.3 | 24.55 | 4.4 | 112.1 | 137.9 | 59.2 |
| 31/03/2017 | 71.7 | 26.35 | 4.8 | 183.1 | 138.3 | 81.0 |
| 01/04/2017 | 11.0 | 25.3 | 4.6 | 190.0 | 138.2 | 81.0 |
| 02/04/2017 | 6.0 | 25 | 4.5 | 194.7 | 138.1 | 81.0 |
| 03/04/2017 | 34.6 | 25.7 | 4.6 | 229.3 | 138.2 | 81.0 |
| 04/04/2017 | 1.2 | 24.8 | 4.5 | 230.5 | 137.9 | 81.0 |
| 05/04/2017 | 0.1 | 26.05 | 4.7 | 230.6 | 138.1 | 81.0 |
| 06/04/2017 | 1.3 | 25.5 | 4.6 | 231.9 | 138.1 | 81.0 |
| 07/04/2017 | 0.0 | 25.85 | 4.7 | 231.9 | 138.2 | 81.0 |
| 08/04/2017 | 1.1 | 25.2 | 4.6 | 224.1 | 138.5 | 81.0 |
| 09/04/2017 | 8.2 | 25.45 | 4.6 | 222.3 | 138.7 | 81.0 |
| 10/04/2017 | 3.5 | 25.45 | 4.6 | 222.3 | 138.8 | 81.0 |
| 11/04/2017 | 0.0 | 24.95 | 4.5 | 215.5 | 138.7 | 81.0 |
| 12/04/2017 | 0.0 | 25.85 | 4.7 | 212.2 | 138.9 | 81.0 |
| 13/04/2017 | 0.0 | 25.7 | 4.6 | 211.8 | 139.0 | 81.0 |
| 14/04/2017 | 0.0 | 25.1 | 4.5 | 207.6 | 138.8 | 81.0 |
| 15/04/2017 | 0.0 | 25.25 | 4.6 | 198.5 | 139.0 | 81.0 |
| 16/04/2017 | 0.0 | 26.1 | 4.7 | 190.3 | 139.1 | 81.0 |
| 17/04/2017 | 0.0 | 26.4 | 4.8 | 190.3 | 139.3 | 81.0 |
| 18/04/2017 | 0.0 | 25.5 | 4.6 | 190.3 | 139.2 | 81.0 |
| 19/04/2017 | 0.0 | 24.9 | 4.5 | 190.3 | 139.3 | 81.0 |
| 20/04/2017 | 0.0 | 26.1 | 4.7 | 189.8 | 139.4 | 81.0 |
| 21/04/2017 | 0.0 | 27.5 | 5.0 | 189.8 | 139.7 | 81.0 |
| 22/04/2017 | 0.8 | 26.35 | 4.8 | 190.6 | 139.9 | 81.0 |
| 23/04/2017 | 12.7 | 26.8 | 4.8 | 203.3 | 140.2 | 81.0 |
| 24/04/2017 | 9.7 | 26 | 4.7 | 213.0 | 139.6 | 81.0 |
| 25/04/2017 | 0.3 | 25.75 | 4.7 | 213.3 | 139.3 | 81.0 |
| 26/04/2017 | 0.0 | 25.75 | 4.7 | 213.3 | 139.2 | 81.0 |
| 27/04/2017 | 0.0 | 25.4 | 4.6 | 166.3 | 139.0 | 81.0 |
| 28/04/2017 | 0.0 | 25.1 | 4.5 | 162.5 | 139.3 | 81.0 |
| 29/04/2017 | 5.0 | 26 | 4.7 | 167.2 | 139.5 | 81.0 |
| 30/04/2017 | 0.0 | 25.35 | 4.6 | 95.5 | 139.3 | 41.2 |
| 01/05/2017 | 0.0 | 24.75 | 4.5 | 84.5 | 139.2 | 30.3 |
| 02/05/2017 | 48.9 | 24.1 | 4.4 | 127.4 | 139.1 | 73.3 |
| 03/05/2017 | 0.8 | 24.05 | 4.4 | 93.6 | 138.8 | 39.8 |
| 04/05/2017 | 8.5 | 24 | 4.3 | 100.9 | 138.6 | 47.3 |
| 05/05/2017 | 5.5 | 25.05 | 4.5 | 106.3 | 138.5 | 52.8 |
| 06/05/2017 | 0.0 | 23.25 | 4.2 | 105.0 | 138.1 | 51.9 |
| 07/05/2017 | 0.1 | 25.05 | 4.5 | 105.1 | 137.9 | 52.2 |
| 08/05/2017 | 1.3 | 24 | 4.3 | 105.3 | 137.7 | 52.6 |
| 09/05/2017 | 0.0 | 24.85 | 4.5 | 97.1 | 137.6 | 44.5 |
| 10/05/2017 | 0.0 | 25.5 | 4.6 | 93.6 | 137.6 | 41.0 |
| 11/05/2017 | 0.0 | 24.9 | 4.5 | 93.6 | 137.6 | 41.0 |
| 12/05/2017 | 0.0 | 23.55 | 4.3 | 93.6 | 137.2 | 41.4 |
| 13/05/2017 | 0.0 | 22.55 | 4.1 | 93.6 | 136.6 | 42.0 |
| 14/05/2017 | 0.0 | 24.55 | 4.4 | 93.6 | 136.5 | 42.1 |
| 15/05/2017 | 0.0 | 25.25 | 4.6 | 93.6 | 136.5 | 42.1 |
| 16/05/2017 | 0.0 | 26.75 | 4.8 | 93.6 | 136.6 | 42.0 |
| 17/05/2017 | 0.0 | 24.65 | 4.5 | 93.6 | 136.3 | 42.3 |
| 18/05/2017 | 0.0 | 24.05 | 4.4 | 93.6 | 136.0 | 42.6 |
| 19/05/2017 | 2.2 | 27.75 | 5.0 | 95.8 | 136.5 | 44.3 |
| 20/05/2017 | 3.2 | 27.05 | 4.9 | 99.0 | 136.7 | 47.3 |
| 21/05/2017 | 14.5 | 26.15 | 4.7 | 113.5 | 136.5 | 62.0 |
| 22/05/2017 | 0.6 | 26 | 4.7 | 113.3 | 136.4 | 61.9 |
| 23/05/2017 | 1.2 | 25.15 | 4.6 | 101.8 | 136.1 | 50.7 |
| 24/05/2017 | 9.7 | 22.5 | 4.1 | 101.8 | 135.5 | 51.3 |
| 25/05/2017 | 7.1 | 24.35 | 4.4 | 108.6 | 135.2 | 58.4 |
| 26/05/2017 | 0.0 | 22.6 | 4.1 | 108.6 | 134.7 | 58.9 |
| 27/05/2017 | 7.6 | 23.75 | 4.3 | 116.2 | 134.4 | 66.8 |
| 28/05/2017 | 1.0 | 23 | 4.2 | 117.2 | 134.0 | 68.2 |
| 29/05/2017 | 2.7 | 22.5 | 4.1 | 114.9 | 133.3 | 66.6 |
| 30/05/2017 | 0.0 | 25.15 | 4.6 | 114.9 | 133.3 | 66.6 |
| 31/05/2017 | 0.0 | 25.05 | 4.5 | 114.9 | 133.4 | 66.5 |
| 01/06/2017 | 0.0 | 23.75 | 4.3 | 66.0 | 133.3 | 17.7 |
| 02/06/2017 | 0.0 | 25.2 | 4.6 | 65.2 | 133.5 | 16.7 |
| 03/06/2017 | 6.4 | 25.95 | 4.7 | 63.1 | 133.9 | 14.2 |
| 04/06/2017 | 9.5 | 25.2 | 4.6 | 67.1 | 133.9 | 18.2 |
| 05/06/2017 | 4.2 | 24.25 | 4.4 | 71.3 | 134.1 | 22.2 |
| 06/06/2017 | 2.2 | 22.55 | 4.1 | 73.4 | 133.6 | 24.8 |
| 07/06/2017 | 4.6 | 23.95 | 4.3 | 76.7 | 133.6 | 28.1 |
| 08/06/2017 | 2.6 | 25.1 | 4.5 | 79.3 | 133.7 | 30.6 |
| 09/06/2017 | 0.0 | 21.2 | 3.8 | 79.3 | 132.9 | 31.4 |
| 10/06/2017 | 0.0 | 24.8 | 4.5 | 79.3 | 132.9 | 31.4 |
| 11/06/2017 | 0.0 | 25 | 4.5 | 79.3 | 133.1 | 31.2 |
| 12/06/2017 | 5.0 | 22 | 4.0 | 84.3 | 133.0 | 36.3 |
| 13/06/2017 | 0.0 | 23 | 4.2 | 84.3 | 132.7 | 36.6 |
| 14/06/2017 | 0.0 | 22.6 | 4.1 | 84.3 | 132.3 | 37.0 |
| 15/06/2017 | 19.0 | 21.55 | 3.9 | 103.3 | 131.3 | 57.0 |
| 16/06/2017 | 0.0 | 20.05 | 3.6 | 103.3 | 130.5 | 57.8 |
| 17/06/2017 | 0.0 | 21.65 | 3.9 | 103.3 | 130.1 | 58.2 |
| 18/06/2017 | 0.0 | 28.85 | 5.2 | 101.1 | 130.3 | 55.8 |
| 19/06/2017 | 2.8 | 19.05 | 3.4 | 100.7 | 128.8 | 56.9 |
| 20/06/2017 | 1.5 | 25.15 | 4.6 | 87.7 | 128.6 | 44.1 |
| 21/06/2017 | 1.6 | 18.15 | 3.3 | 88.7 | 127.2 | 46.5 |
| 22/06/2017 | 22.1 | 21.6 | 3.9 | 109.6 | 126.6 | 68.0 |
| 23/06/2017 | 16.0 | 26.25 | 4.7 | 115.9 | 127.2 | 73.7 |
| 24/06/2017 | 4.1 | 22 | 4.0 | 112.9 | 126.8 | 71.1 |
| 25/06/2017 | 3.1 | 21.7 | 3.9 | 116.0 | 126.7 | 74.3 |
| 26/06/2017 | 5.8 | 24.9 | 4.5 | 114.2 | 126.9 | 72.3 |
| 27/06/2017 | 16.0 | 22.55 | 4.1 | 129.2 | 126.8 | 81.0 |
| 28/06/2017 | 11.4 | 23.4 | 4.2 | 137.9 | 126.9 | 81.0 |
| 29/06/2017 | 3.1 | 23.4 | 4.2 | 141.0 | 126.6 | 81.0 |
| 30/06/2017 | 2.4 | 23.05 | 4.2 | 143.4 | 126.3 | 81.0 |
| 01/07/2017 | 0.0 | 22.75 | 4.1 | 143.4 | 126.1 | 81.0 |
| 02/07/2017 | 0.2 | 25 | 4.5 | 143.6 | 126.0 | 81.0 |
| 03/07/2017 | 0.4 | 19.5 | 3.5 | 137.6 | 124.9 | 81.0 |
| 04/07/2017 | 3.7 | 20.15 | 3.6 | 131.8 | 124.0 | 81.0 |
| 05/07/2017 | 8.8 | 21 | 3.8 | 136.4 | 123.4 | 81.0 |
| 06/07/2017 | 3.0 | 22.05 | 4.0 | 137.2 | 123.3 | 81.0 |
| 07/07/2017 | 3.8 | 19.3 | 3.5 | 136.4 | 122.4 | 81.0 |
| 08/07/2017 | 8.1 | 20.55 | 3.7 | 141.9 | 121.6 | 81.0 |
| 09/07/2017 | 69.2 | 19.95 | 3.6 | 211.1 | 121.4 | 81.0 |
| 10/07/2017 | 2.3 | 21.6 | 3.9 | 213.4 | 120.8 | 81.0 |
| 11/07/2017 | 6.5 | 21.4 | 3.9 | 219.9 | 120.2 | 81.0 |
| 12/07/2017 | 3.9 | 20.95 | 3.8 | 218.8 | 120.0 | 81.0 |
| 13/07/2017 | 5.7 | 21.65 | 3.9 | 224.5 | 119.7 | 81.0 |
| 14/07/2017 | 13.6 | 20.35 | 3.7 | 238.1 | 119.3 | 81.0 |
| 15/07/2017 | 2.4 | 24.4 | 4.4 | 221.5 | 119.8 | 81.0 |
| 16/07/2017 | 17.4 | 20.4 | 3.7 | 238.9 | 119.9 | 81.0 |
| 17/07/2017 | 4.8 | 21.5 | 3.9 | 243.7 | 119.9 | 81.0 |
| 18/07/2017 | 0.2 | 19.25 | 3.5 | 243.9 | 118.1 | 81.0 |
| 19/07/2017 | 10.7 | 20 | 3.6 | 251.8 | 118.3 | 81.0 |
| 20/07/2017 | 1.6 | 21.55 | 3.9 | 251.9 | 117.7 | 81.0 |
| 21/07/2017 | 0.0 | 22.85 | 4.1 | 250.3 | 118.5 | 81.0 |
| 22/07/2017 | 0.0 | 22.2 | 4.0 | 228.2 | 118.6 | 81.0 |
| 23/07/2017 | 7.2 | 26.05 | 4.7 | 219.4 | 118.6 | 81.0 |
| 24/07/2017 | 19.4 | 21.25 | 3.8 | 234.7 | 118.4 | 81.0 |
| 25/07/2017 | 1.5 | 21.55 | 3.9 | 233.1 | 118.4 | 81.0 |
| 26/07/2017 | 1.6 | 20.45 | 3.7 | 228.9 | 117.6 | 81.0 |
| 27/07/2017 | 3.8 | 21.4 | 3.9 | 216.7 | 117.4 | 81.0 |
| 28/07/2017 | 12.0 | 22.05 | 4.0 | 217.3 | 117.2 | 81.0 |
| 29/07/2017 | 4.1 | 27.6 | 5.0 | 218.3 | 117.9 | 81.0 |
| 30/07/2017 | 10.0 | 23.5 | 4.3 | 225.9 | 118.0 | 81.0 |
| 31/07/2017 | 3.5 | 20.65 | 3.7 | 229.4 | 117.6 | 81.0 |
| 01/08/2017 | 0.1 | 21.05 | 3.8 | 229.3 | 116.9 | 81.0 |
| 02/08/2017 | 0.0 | 22.4 | 4.1 | 228.9 | 117.4 | 81.0 |
| 03/08/2017 | 0.0 | 23.35 | 4.2 | 225.2 | 118.0 | 81.0 |
| 04/08/2017 | 0.0 | 22.65 | 4.1 | 216.4 | 118.3 | 81.0 |
| 05/08/2017 | 5.6 | 24.55 | 4.4 | 219.0 | 118.8 | 81.0 |
| 06/08/2017 | 2.9 | 23.55 | 4.3 | 218.1 | 119.5 | 81.0 |
| 07/08/2017 | 0.0 | 21.6 | 3.9 | 210.0 | 119.7 | 81.0 |
| 08/08/2017 | 0.0 | 20.8 | 3.8 | 140.8 | 119.9 | 81.0 |
| 09/08/2017 | 0.0 | 21.25 | 3.8 | 138.5 | 119.8 | 81.0 |
| 10/08/2017 | 0.0 | 24.35 | 4.4 | 132.0 | 120.3 | 81.0 |
| 11/08/2017 | 0.0 | 23.9 | 4.3 | 128.1 | 120.9 | 81.0 |
| 12/08/2017 | 0.0 | 21.45 | 3.9 | 122.4 | 120.8 | 81.0 |
| 13/08/2017 | 0.0 | 23.65 | 4.3 | 108.8 | 121.4 | 72.4 |
| 14/08/2017 | 0.0 | 20 | 3.6 | 106.4 | 120.6 | 70.8 |
| 15/08/2017 | 0.9 | 21.8 | 3.9 | 89.9 | 120.9 | 54.0 |
| 16/08/2017 | 1.0 | 19.1 | 3.5 | 86.1 | 120.5 | 50.6 |
| 17/08/2017 | 0.0 | 21.7 | 3.9 | 85.9 | 120.9 | 50.0 |
| 18/08/2017 | 0.0 | 21.5 | 3.9 | 75.2 | 121.2 | 39.0 |
| 19/08/2017 | 0.0 | 25.65 | 4.6 | 73.6 | 121.9 | 36.7 |
| 20/08/2017 | 0.0 | 22.85 | 4.1 | 73.6 | 121.9 | 36.7 |
| 21/08/2017 | 11.4 | 23.55 | 4.3 | 85.0 | 122.2 | 47.8 |
| 22/08/2017 | 6.0 | 23 | 4.2 | 83.8 | 121.6 | 47.2 |
| 23/08/2017 | 1.0 | 23.05 | 4.2 | 65.4 | 121.9 | 28.5 |
| 24/08/2017 | 7.2 | 21.6 | 3.9 | 71.1 | 121.9 | 34.2 |
| 25/08/2017 | 9.5 | 20.15 | 3.6 | 79.0 | 121.9 | 42.1 |
| 26/08/2017 | 1.2 | 21 | 3.8 | 76.4 | 121.8 | 39.6 |
| 27/08/2017 | 6.8 | 21.05 | 3.8 | 71.2 | 121.6 | 34.6 |
| 28/08/2017 | 0.0 | 21.85 | 4.0 | 67.1 | 120.6 | 31.5 |
| 29/08/2017 | 0.0 | 22.15 | 4.0 | 57.1 | 120.4 | 21.7 |
| 30/08/2017 | 0.0 | 23 | 4.2 | 53.6 | 120.8 | 17.8 |
| 31/08/2017 | 0.0 | 21.65 | 3.9 | 53.5 | 120.9 | 17.6 |
| 01/09/2017 | 0.0 | 23.45 | 4.2 | 53.5 | 121.1 | 17.4 |
| 02/09/2017 | 26.0 | 19.15 | 3.5 | 79.5 | 120.3 | 44.2 |
| 03/09/2017 | 7.9 | 22.5 | 4.1 | 87.4 | 120.3 | 52.1 |
| 04/09/2017 | 1.0 | 21.3 | 3.9 | 82.8 | 119.7 | 48.1 |
| 05/09/2017 | 0.0 | 21.2 | 3.8 | 79.9 | 119.3 | 45.6 |
| 06/09/2017 | 0.0 | 24.5 | 4.4 | 79.9 | 119.8 | 45.1 |
| 07/09/2017 | 28.1 | 23 | 4.2 | 108.0 | 120.2 | 72.8 |
| 08/09/2017 | 1.7 | 28.05 | 5.1 | 109.7 | 121.4 | 73.3 |
| 09/09/2017 | 1.4 | 23.45 | 4.2 | 111.1 | 121.3 | 74.8 |
| 10/09/2017 | 6.3 | 21.1 | 3.8 | 117.4 | 120.8 | 81.0 |
| 11/09/2017 | 2.1 | 24.4 | 4.4 | 119.5 | 121.3 | 81.0 |
| 12/09/2017 | 17.5 | 20.15 | 3.6 | 137.0 | 120.7 | 81.0 |
| 13/09/2017 | 20.5 | 21 | 3.8 | 157.5 | 120.8 | 81.0 |
| 14/09/2017 | 1.5 | 23.1 | 4.2 | 158.1 | 121.1 | 81.0 |
| 15/09/2017 | 5.1 | 26.65 | 4.8 | 162.2 | 122.4 | 81.0 |
| 16/09/2017 | 0.3 | 20.1 | 3.6 | 162.5 | 122.2 | 81.0 |
| 17/09/2017 | 0.5 | 22.5 | 4.1 | 163.0 | 122.3 | 81.0 |
| 18/09/2017 | 3.8 | 22.75 | 4.1 | 166.8 | 121.8 | 81.0 |
| 19/09/2017 | 2.9 | 22.25 | 4.0 | 169.7 | 121.7 | 81.0 |
| 20/09/2017 | 5.5 | 21.65 | 3.9 | 163.8 | 121.4 | 81.0 |
| 21/09/2017 | 1.0 | 23.25 | 4.2 | 158.8 | 121.4 | 81.0 |
| 22/09/2017 | 3.8 | 20.75 | 3.8 | 161.6 | 121.0 | 81.0 |
| 23/09/2017 | 1.7 | 20.7 | 3.7 | 156.1 | 120.8 | 81.0 |
| 24/09/2017 | 0.7 | 21.8 | 3.9 | 147.3 | 121.1 | 81.0 |
| 25/09/2017 | 5.6 | 21.75 | 3.9 | 151.7 | 121.3 | 81.0 |
| 26/09/2017 | 9.9 | 21.7 | 3.9 | 154.8 | 121.4 | 81.0 |
| 27/09/2017 | 3.5 | 24.3 | 4.4 | 158.3 | 121.8 | 81.0 |
| 28/09/2017 | 0.0 | 24.55 | 4.4 | 158.3 | 122.3 | 81.0 |
| 29/09/2017 | 0.0 | 21.65 | 3.9 | 158.3 | 122.0 | 81.0 |
| 30/09/2017 | 0.0 | 23.05 | 4.2 | 158.3 | 122.3 | 81.0 |
| 01/10/2017 | 0.0 | 24.95 | 4.5 | 158.3 | 122.5 | 81.0 |
| 02/10/2017 | 0.0 | 24.35 | 4.4 | 132.3 | 123.5 | 81.0 |
| 03/10/2017 | 0.0 | 23.6 | 4.3 | 124.4 | 123.7 | 81.0 |
| 04/10/2017 | 6.5 | 24.55 | 4.4 | 129.9 | 124.3 | 81.0 |
| 05/10/2017 | 1.2 | 23.5 | 4.3 | 131.1 | 124.7 | 81.0 |
| 06/10/2017 | 1.6 | 22.1 | 4.0 | 132.7 | 124.2 | 81.0 |
| 07/10/2017 | 18.2 | 22.65 | 4.1 | 122.8 | 124.2 | 81.0 |
| 08/10/2017 | 4.3 | 23.55 | 4.3 | 125.4 | 123.4 | 81.0 |
| 09/10/2017 | 0.0 | 23.15 | 4.2 | 124.0 | 123.3 | 81.0 |
| 10/10/2017 | 0.1 | 21.7 | 3.9 | 117.8 | 123.4 | 79.4 |
| 11/10/2017 | 0.0 | 25.1 | 4.5 | 115.7 | 123.5 | 77.2 |
| 12/10/2017 | 6.3 | 25 | 4.5 | 104.5 | 124.4 | 65.1 |
| 13/10/2017 | 9.4 | 22.95 | 4.2 | 93.4 | 124.8 | 53.6 |
| 14/10/2017 | 2.0 | 29.05 | 5.3 | 93.9 | 125.9 | 53.0 |
| 15/10/2017 | 0.1 | 25 | 4.5 | 88.9 | 125.6 | 48.3 |
| 16/10/2017 | 0.0 | 22.95 | 4.2 | 88.6 | 126.1 | 47.5 |
| 17/10/2017 | 0.0 | 23.5 | 4.3 | 88.1 | 126.3 | 46.8 |
| 18/10/2017 | 26.1 | 23 | 4.2 | 110.4 | 126.3 | 69.1 |
| 19/10/2017 | 0.0 | 23 | 4.2 | 107.5 | 126.4 | 66.1 |
| 20/10/2017 | 1.7 | 23.5 | 4.3 | 103.7 | 126.8 | 61.9 |
| 21/10/2017 | 0.4 | 24.55 | 4.4 | 103.1 | 127.0 | 61.1 |
| 22/10/2017 | 0.2 | 25.5 | 4.6 | 99.5 | 127.9 | 56.6 |
| 23/10/2017 | 0.0 | 23.65 | 4.3 | 97.8 | 128.4 | 54.4 |
| 24/10/2017 | 1.9 | 23.7 | 4.3 | 99.0 | 128.7 | 55.3 |
| 25/10/2017 | 0.0 | 24.2 | 4.4 | 93.4 | 129.2 | 49.2 |
| 26/10/2017 | 1.3 | 23.15 | 4.2 | 84.8 | 129.4 | 40.4 |
| 27/10/2017 | 0.8 | 24.05 | 4.4 | 82.1 | 129.4 | 37.7 |
| 28/10/2017 | 0.0 | 24.55 | 4.4 | 82.1 | 129.4 | 37.7 |
| 29/10/2017 | 0.0 | 23.45 | 4.2 | 82.1 | 129.7 | 37.4 |
| 30/10/2017 | 0.0 | 25.25 | 4.6 | 82.1 | 130.1 | 37.0 |
| 31/10/2017 | 0.0 | 24.75 | 4.5 | 82.1 | 130.1 | 37.0 |
| 01/11/2017 | 61.1 | 24.7 | 4.5 | 143.2 | 130.2 | 81.0 |
| 02/11/2017 | 19.5 | 25.2 | 4.6 | 162.7 | 130.4 | 81.0 |
| 03/11/2017 | 0.3 | 24.8 | 4.5 | 156.5 | 130.5 | 81.0 |
| 04/11/2017 | 1.4 | 24.05 | 4.4 | 156.7 | 130.6 | 81.0 |
| 05/11/2017 | 3.2 | 26.5 | 4.8 | 158.3 | 131.4 | 81.0 |
| 06/11/2017 | 9.1 | 24.5 | 4.4 | 149.2 | 131.7 | 81.0 |
| 07/11/2017 | 0.0 | 24.1 | 4.4 | 144.9 | 131.8 | 81.0 |
| 08/11/2017 | 0.0 | 25.5 | 4.6 | 144.9 | 132.2 | 81.0 |
| 09/11/2017 | 0.7 | 25.5 | 4.6 | 145.5 | 132.9 | 81.0 |
| 10/11/2017 | 1.7 | 23.2 | 4.2 | 147.2 | 132.6 | 81.0 |
| 11/11/2017 | 0.0 | 25.55 | 4.6 | 140.9 | 132.7 | 81.0 |
| 12/11/2017 | 28.0 | 26 | 4.7 | 159.5 | 133.2 | 81.0 |
