# Supplementary material for: Climate change could threaten cocoa production: Effects of 2015-16 El Niño-related drought on cocoa agroforests in Bahia, Brazil
Source: PLoS One. 2018 Jul 10;13(7):e0200454. doi: 10.1371/journal.pone.0200454 (PMC6039034; doi:10.1371/journal.pone.0200454)
Supplement: S3 Table — (DOCX) [file pone.0200454.s003.docx]

**S3 Table**. Soil water holding capacity in 10 cocoa farms, Barro Preto

| farm ID |  | water in soil (mm) | | |  | farm location | | |
| --- | --- | --- | --- | --- | --- | --- | --- | --- |
|  | 0-20 cm | 20-40 cm | 40-60 cm | 0-60 cm | | longitude |  | |
| 1 | 13.51 | 8.94 | 7.27 | | 29.7 | -39.3798 | | East |
| 2 | 4.19 | - | - | | - | -39.5190 | | West |
| 3 | 16.11 | 24.13 | 17.78 | | 58.0 | -39.4814 | | West |
| 4 | 15.67 | 8.36 | - | | 24.0 | -39.3626 | | East |
| 5 | 17.8 | 10.82 | 7.82 | | 36.4 | -39.4796 | | West |
| 6 | 12.69 | 27.62 | 30.11 | | 70.4 | -39.498 | | West |
| 7 | 27.86 | 23.89 | 24.35 | | 76.1 | -39.5223 | | West |
| 8 | 12.36 | 13.31 | 3.37 | | 29.0 | -39.3865 | | East |
| 9 | 14.05 | 18.48 | 22.27 | | 54.8 | -39.5214 | | West |
| 10 | 17.13 | 19.79 | 21.4 | | 58.3 | -39.4692 | | West |
