# Supplementary material for: Climate change could threaten cocoa production: Effects of 2015-16 El Niño-related drought on cocoa agroforests in Bahia, Brazil
Source: PLoS One. 2018 Jul 10;13(7):e0200454. doi: 10.1371/journal.pone.0200454 (PMC6039034; doi:10.1371/journal.pone.0200454)
Supplement: S4 Table — (DOCX) [file pone.0200454.s004.docx]

**S4 Table.** Tree mortality per cocoa farms after 2015-16 ENSO drought

| Farm ID | total cocoa trees per transect | dead cocoa trees per transect | half-dead cocoa trees per transect | mortality (*m*) |
| --- | --- | --- | --- | --- |
| 1 | 40 | 1 | 4 | 0.03 |
| 2 | 41 | 0 | 1 | 0.00 |
| 3 | 36 | 10 | 8 | 0.28 |
| 4 | 45 | 36 | 5 | 0.80 |
| 5 | 59 | 5 | 2 | 0.08 |
| 6 | 61 | 6 | 3 | 0.10 |
| 7 | 68 | 9 | 2 | 0.13 |
| 8 | 57 | 3 | 2 | 0.05 |
| 9 | 64 | 5 | 3 | 0.08 |
| 10 | 65 | 8 | 1 | 0.12 |
| 11 | 64 | 15 | 6 | 0.23 |
| 12 | 41 | 8 | 0 | 0.20 |
| 13 | 33 | 0 | 1 | 0.00 |
| 14 | 44 | 24 | 10 | 0.55 |
| 15 | 68 | 7 | 0 | 0.10 |
| 16 | 40 | 5 | 1 | 0.13 |
| 17 | 31 | 4 | 1 | 0.13 |
| 18 | 45 | 5 | 3 | 0.11 |
| 19 | 51 | 1 | 2 | 0.02 |
| 20 | 65 | 3 | 3 | 0.05 |
| 21 | 39 | 4 | 0 | 0.10 |
| 22 | 25 | 0 | 0 | 0.00 |
| 23 | 59 | 5 | 0 | 0.08 |
| 24 | 63 | 26 | 9 | 0.41 |
| 25 | 39 | 2 | 3 | 0.05 |
| 26 | 50 | 0 | 1 | 0.00 |
| 27 | 55 | 7 | 12 | 0.13 |
| 28 | 51 | 0 | 2 | 0.00 |
| 29 | 44 | 7 | 1 | 0.16 |
| 30 | 70 | 6 | 0 | 0.08 |
| 31 | 98 | 33 | 5 | 0.34 |
