# Supplementary material for: Climate change could threaten cocoa production: Effects of 2015-16 El Niño-related drought on cocoa agroforests in Bahia, Brazil
Source: PLoS One. 2018 Jul 10;13(7):e0200454. doi: 10.1371/journal.pone.0200454 (PMC6039034; doi:10.1371/journal.pone.0200454)
Supplement: S5 Table — (DOCX) [file pone.0200454.s005.docx]

**S5 Table.** Yield in kg/ha per farm following five peak harvests: before (April 2015), during (April and November 2016) and after (April and November 2017) 2015-16 ENSO drought.

| farm ID | before ENSO | during ENSO | | | after ENSO | | |
| --- | --- | --- | --- | --- | --- | --- | --- |
|  | Apr-15 | Apr-16 | | Nov-16 | Apr-17 | | Nov-17 |
| 1 | 389.5 | 21.9 | 76.2 | | 31 | 45 | |
| 2 | 72.5 | 10.6 | 24.4 | | 31.5 | 71.5 | |
| 3 | 127 | 3.5 | 15.2 | | 13 | 23.5 | |
| 4 | 63 | 0.0 | 8.3 | | 0.5 | 4.5 | |
| 5 | 496 | 179.4 | 19.6 | | 19.5 | 37 | |
| 6 | 203 | 0.9 | 42.8 | | 35.5 | 27 | |
| 7 | 432.5 | 0.0 | 182.6 | | 126.3 | 307.5 | |
| 8 | 155.5 | 7.8 | 53.2 | | 13.5 | 24.5 | |
| 9 | 371 | 65.3 | 90.6 | | 49 | 87.5 | |
| 10 | 271.5 | 23.6 | 50.6 | | 17.5 | 110 | |
| 11 | 83.5 | 1.4 | 21.3 | | 3.5 | 18.5 | |
| 12 | 263 | 20.1 | 18.7 | | 22.5 | 23 | |
| 13 | 515.5 | 2.6 | 52.9 | | 21 | 31.5 | |
| 14 | 102.5 | 8.3 | 29.3 | | 29.5 | 28.5 | |
| 15 | 185.5 | 4.6 | 280.0 | | 119 | 25.5 | |
| 16 | 81 | 11.5 | 11.8 | | 43.5 | 197 | |
| 17 | 348.5 | 11.8 | 55.8 | | 24.5 | 124.5 | |
| 18 | 186.5 | 0.0 | 59.2 | | 24.5 | 120.5 | |
| 19 | 69 | 40.3 | 40.3 | | 116.5 | 90 | |
| 20 | 514 | 22.7 | 19.3 | | 2 | 35 | |
| 21 | 162 | 44.0 | 68.4 | | 33 | 36.5 | |
| 22 | 134.5 | 55.5 | 18.7 | | 23 | 18 | |
| 23 | 363 | 83.4 | 48.9 | | 68.5 | 59.5 | |
| 24 | 80 | 2.9 | 28.5 | | 3 | 41 | |
| 25 | 205.5 | 25.3 | 34.8 | | 37 | 28 | |
| 26 | 200 | 30.8 | 74.2 | | 113.5 | 106.5 | |
| 27 | 125.5 | 2.6 | 17.3 | | 4.5 | 11.5 | |
| 28 | 486.5 | 67.9 | 8.3 | | 46 | 26.5 | |
| 29 | 386 | 30.8 | 96.0 | | 82 | 62.5 | |
| 30 | 324.5 | 19.3 | 102.9 | | 62 | 116.5 | |
| 31 | 101.5 | 0.0 | 27.9 | | 21.5 | 39.5 | |
