# Supplementary material for: Climate change could threaten cocoa production: Effects of 2015-16 El Niño-related drought on cocoa agroforests in Bahia, Brazil
Source: PLoS One. 2018 Jul 10;13(7):e0200454. doi: 10.1371/journal.pone.0200454 (PMC6039034; doi:10.1371/journal.pone.0200454)
Supplement: S6 Table — (DOCX) [file pone.0200454.s006.docx]

**S6 Table.** Harvest of ripe pods for conversion factors fresh weight into dry weight

| May-12 | number of fruits | total fresh pod (g) | fresh husk + resides (g) | fresh beans (g) | Total dry beans (g) | dry beans /pod (g) |
| --- | --- | --- | --- | --- | --- | --- |
| plot 1 | 5 | 2857.1 | 2001.5 | 861.5 | 333.4 | 66.7 |
| plot 2 | 5 | 2599.1 | 2014 | 570.8 | 235.5 | 47.1 |
| plot 3 | 5 | 2596.7 | 2088.7 | 523.2 | 152 | 30.4 |
| plot 4 | 5 | 2540.4 | 2042.2 | 492.8 | 186.4 | 37.3 |
| plot 5 | 5 | 1710.8 | 1355 | 365.5 | 155 | 31.0 |
| plot 6 | 5 | 2288.1 | 2177.1 | 431.2 | 145.8 | 29.2 |
| average dry fermented bean weight | | | |  |  | 40.3 |
|  |  |  |  |  |  |  |
| May-16 | number of fruits | total fresh pod (g) | fresh husk + residues (g) | fresh beans (g) | Total dry beans (g) | dry beans /pod (g) |
| Farm 1 | 8 | 2804.2 | 2104.7 | 783.8 | 210.2 | 26.3 |
| Farm 2 | 10 | 2585.9 | 1773.7 | 822.3 | 316.2 | 31.6 |
| Farm 3 | 8 | 2069.6 | 1625.2 | 445.1 | 130.1 | 16.3 |
| Farm 4 | 8 | 2273.7 | 1602.2 | 697.1 | 144.0 | 18.0 |
| average dry fermented bean weight | | | |  |  | 23.0 |
