# Supplementary material for: Climate change could threaten cocoa production: Effects of 2015-16 El Niño-related drought on cocoa agroforests in Bahia, Brazil
Source: PLoS One. 2018 Jul 10;13(7):e0200454. doi: 10.1371/journal.pone.0200454 (PMC6039034; doi:10.1371/journal.pone.0200454)
Supplement: S7 Table — (DOCX) [file pone.0200454.s007.docx]

**S7 Table**. Farm variables description

| farm | unit | n | mean | SE |
| --- | --- | --- | --- | --- |
| SumDBHShade | cm^2^/800 m^2^ | 31 | 335.5 | 26.5 |
| NumShade | tree/800 m^2^ | 31 | 9.9 | 1.0 |
| CocoaTreeDensity | tree/800 m^2^ | 31 | 51.9 | 2.7 |
| PerDead | % | 31 | 13.4 | 2.9 |
| longitude | degree west | 31 | 39.456 | 0.010 |
| GrndCover | % | 30 | 42.3 | 2.1 |
| Water In soil | mm | 9 | 48.5 | 6.4 |
| differenceInYield | pods/800 m^2^ | 31 | 147.8 | 29.0 |
