# Supplementary material for: Climate change could threaten cocoa production: Effects of 2015-16 El Niño-related drought on cocoa agroforests in Bahia, Brazil
Source: PLoS One. 2018 Jul 10;13(7):e0200454. doi: 10.1371/journal.pone.0200454 (PMC6039034; doi:10.1371/journal.pone.0200454)
Supplement: S8 Table — (DOCX) [file pone.0200454.s008.docx]

**S8 Table**. Cocoa production in Brazil since 1990 (source FAO and IBGE)

| **year** | Brazilian cocoa | Brazil | Bahia | Pará | Barro Preto |
| --- | --- | --- | --- | --- | --- |
|  | production total (t) |  | cocoa productivity (kg/ha) | | |
| 1990 | 356246 | 535.8 | 543.4 | 593.7 | 350.0 |
| 1991 | 320967 | 480.6 | 477.0 | 544.4 | 350.0 |
| 1992 | 328518 | 449.1 | 433.1 | 572.9 | 450.0 |
| 1993 | 340885 | 464.3 | 457.1 | 597.8 | 500.0 |
| 1994 | 330577 | 473.2 | 467.0 | 637.6 | 500.0 |
| 1995 | 296705 | 401.7 | 393.9 | 554.1 | 390.0 |
| 1996 | 256777 | 387.9 | 353.9 | 668.0 | 400.0 |
| 1997 | 277966 | 388.4 | 360.3 | 615.4 | 450.0 |
| 1998 | 280801 | 395.6 | 378.3 | 680.0 | 450.0 |
| 1999 | 205003 | 301.0 | 266.5 | 728.8 | 228.5 |
| 2000 | 196788 | 278.8 | 226.7 | 727.3 | 157.0 |
| 2001 | 185662 | 278.9 | 220.7 | 750.5 | 134.9 |
| 2002 | 174796 | 300.2 | 225.9 | 820.2 | 41.0 |
| 2003 | 170004 | 287.7 | 227.0 | 614.7 | 41.5 |
| 2004 | 196005 | 306.8 | 254.5 | 644.5 | 41.0 |
| 2005 | 208620 | 333.6 | 265.6 | 736.9 | 188.3 |
| 2006 | 212270 | 328.0 | 275.4 | 636.9 | 193.7 |
| 2007 | 201651 | 320.6 | 260.0 | 671.7 | 196.6 |
| 2008 | 202030 | 315.0 | 251.7 | 689.6 | 202.4 |
| 2009 | 218487 | 343.5 | 268.4 | 771.4 | 182.7 |
| 2010 | 235389 | 356.3 | 285.1 | 728.2 | 210.0 |
| 2011 | 248524 | 365.2 | 293.1 | 750.2 | 250.0 |
| 2012 | 253211 | 370.0 | 299.6 | 762.4 | 241.5 |
| 2013 | 256186 | 371.7 | 286.7 | 820.4 | 183.0 |
| 2014 | 273793 | 388.8 | 294.3 | 860.6 | 183.3 |
| 2015 | 273124 | 403.5 | 298.3 | 858.6 | 181.3 |
| 2016 | 214698 | 276.5 | 207.3 | 490.0 | 131.8 |
| 2017 | 235526 | 307.2 | 190.9 | 664.3 | - |
