# Supplementary material for: Climate change could threaten cocoa production: Effects of 2015-16 El Niño-related drought on cocoa agroforests in Bahia, Brazil
Source: PLoS One. 2018 Jul 10;13(7):e0200454. doi: 10.1371/journal.pone.0200454 (PMC6039034; doi:10.1371/journal.pone.0200454)
Supplement: S9 Table — (DOCX) [file pone.0200454.s009.docx]

**S9 Table.** List of shade trees species found in 2.5 ha (31 farms) in Barro Preto, Bahia, Brazil

| Number of individual | specie | uses | | IUCN statue | native | evergreen or deciduous |
| --- | --- | --- | --- | --- | --- | --- |
| 331 | *Musa spp* | fruit | |  |  | evergreen |
| 42 | *Artocarpus heterophilus* | fruit | |  |  | deciduous |
| 19 | *Ficus spp* | environmental | |  |  | deciduous |
| 18 | *Citrus sinensis* | fruit | |  |  | evergreen |
| 18 | *Cordia spp* | timber | |  |  | deciduous |
| 15 | *Plathymenia foliosa* | timber, N-fixing | | vulnerable | Atlantic forest | deciduous |
| 12 | *Spondias mombin* | fruit | |  |  | deciduous |
| 11 | *Coffea spp* | fruit | |  |  | evergreen |
| 11 | *Erythrina spp* | N-fixing | |  |  | deciduous |
| 11 | *Genipa americana* | fruit | |  |  | deciduous |
| 11 | *Lonchocarpus glabrescens* | timber, N-fixing | | vulnerable | Atlantic forest | ?  ? |
| 9 | *Senna multijuga* | N-fixing | |  | Atlantic forest | ? |
| 9 | *Trema micrantha* | timber | | vulnerable | Atlantic forest | ? |
| 8 | *Cedrela spp* | timber | | Vulnerable A1cd+2cd | Atlantic forest | deciduous |
| 8 | *Inga spp* | fruit, timber, N-fixing | |  |  | evergreen |
| 7 | *Persea americana* | fruit | |  |  | evergreen |
| 6 | *Carica papaya* | fruit | |  |  | evergreen |
| 6 | *Caryocar brasiliense* | fruit | | nd | Cerrado | ? |
| 6 | *Citrus reticulata* | fruit | |  |  | evergreen |
| 6 | *Erythrina poeppigiana* | N-fixing | | nd |  | deciduous |
| 5 | *Clitoria fairchildiana* | N-fixing | | nd |  | winter-deciduous |
| 5 | *Jacaranda puberula* | timber | | Vulnerable B1+2ac | Atlantic forest | deciduous |
| 4 | *Andira anthelmia* | medicinal, N-fixing | | Least Concern ver 3.1 | Atlantic forest | ? |
| 4 | *Bactris gasipaes* | heart palm | |  |  | evergreen |
| 4 | *Cecropia lyratiloba* | timber | | nd |  | semi-deciduous |
| 4 | *Cordia superba* | timber | |  | Atlantic forest | deciduous |
| 4 | *Erythrina glauca* | N-fixing | | nd |  | deciduous |
| 4 | *Euterpe oleracea* | fruit | |  |  | ? |
| 4 | *Gallesia integrifolia* | timber, medicinal | |  | Atlantic forest | ? |
| 4 | *Tabebuia spp* | timber | |  | Atlantic forest | ? |
| 3 | *Annona reticulata* | fruit | | nd |  | deciduous |
| 3 | *Tapirira guianensis* | timber, medicinal | |  | Atlantic forest | ? |
| 3 | *Macrosyphonia velame* | timber | |  | Atlantic forest | ? |
| 3 | *Mangifera indica* | fruit | |  |  | evergreen |
| 3 | *Pithecolobium polycephalum/Albizia polycephala* | timber, medicinal, N-fixing | | | Atlantic forest | ? |
| 2 | *Aegiphila sellowiana* | timber, medicinal | nd | | Atlantic forest | ? |
| 2 | *Bauhinia fortificata* | medicinal | Least Concern ver 3.1 | | Atlantic forest | ? |
| 2 | *Campomanesia guazumifolia* | fruit | nd | | Atlantic forest | ? |
| 2 | *Cariniana legalis* | timber | Vulnerable A1ac | | Atlantic forest | ? |
| 2 | *Citrus spp* | fruit |  | |  | evergreen |
| 2 | *Eriotheca macrophylla/Bombax sclerophyllum* | timber |  | | Atlantic forest | ? |
| 2 | *Hevea brasiliensis* | rubber |  | |  | deciduous |
| 2 | *Psidium guajava* | fruit |  | |  | evergreen |
| 2 | *Senefeldera verticillata* | timber |  | | Atlantic forest | ? |
| 2 | *unknown* |  |  | |  | ? |
| 1 | *Albizia niopoides* | timber, N-fixing | nd | | Atlantic forest | deciduous |
| 1 | *Citharexylum myrianthum* | fruit |  | | Atlantic forest | ? |
| 1 | *Citrus Limonium* | fruit |  | |  | evergreen |
| 1 | *Cordia elaeagnoides* | timber |  | |  | deciduous |
| 1 | *Cordia trichotoma* | timber |  | |  | deciduous |
| 1 | *Dalbergia nigra* | timber, N-fixing | Vulnerable A1cd | | Atlantic forest | deciduous |
| 1 | *Eugenia florida DC* | fruit | nd | |  | ? |
| 1 | *Eugenia uniflora* | fruit |  | |  | ? |
| 1 | *Handroanthus impetiginosus* | timber, medicinal |  | | Atlantic forest | ? |
| 1 | *Heliconia spp* | flower | nd | |  | evergreen |
| 1 | *Jacaranda cuspidifolia* | timber |  | |  | deciduous |
| 1 | *Matayba eleagnoides* | timber |  | | Atlantic forest | ? |
| 1 | *Myrcia citrifolia* |  |  | | Atlantic forest | ? |
| 1 | *Myrcia spp* |  |  | | Atlantic forest | ? |
| 1 | *Schizolobium parahyba* | timber |  | |  | deciduous |
| 1 | *Psidium myrsinites* | medicinal, fruit |  | |  | evergreen |
| 1 | *Pterocarpus rohrii* | timber, N-fixing |  | | Atlantic forest | ? |
| 1 | *Schefflera morototoni* | timber |  | | Atlantic forest | evergreen |
| 1 | *Swartzia apetala* | timber, N-fixing | Least Concern ver 3.1 | | Atlantic forest | ? |
| 1 | *Syzygium jambolanum* | medicinal |  | | Atlantic forest | ? |
| 1 | *Terminalia kuhlmannii* | timber | Vulnerable D2 | | Atlantic forest | ? |
| 1 | *Theobroma grandiflorum* | fruit |  | |  | evergreen |
| 1 | *Xylopia aromatica* | spices |  | |  | ? |
